# Plant gene silencing signals move from the phloem to influence gene expression in shoot apical meristems

**DOI:** 10.1186/s12870-022-03998-8

**Published:** 2022-12-23

**Authors:** Mark A. A. Minow, Viktoriya Coneva, Victoria Lesy, Max Misyura, Joseph Colasanti

**Affiliations:** Department of Molecular and Cellular Biology, University of Guelph, 50 Stone Road East Guelph, Ontario, Canada

**Keywords:** Arabidopsis, Long-distance signaling, Phloem, Shoot apical meristem (SAM), Small RNA, CLAVATA3, FD, Flowering

## Abstract

**Background:**

Small RNAs (sRNA) are potent regulators of gene expression that can diffuse short distances between cells and move long distances through plant vasculature. However, the degree to which sRNA silencing signals can move from the phloem to the shoot apical meristem (SAM) remains unclear.

**Results:**

Two independent transgenic approaches were used to examine whether phloem sRNA silencing can reach different domains of the SAM and silence SAM-expressed genes. First, the phloem companion-cell specific *SUCROSE-PROTON SYMPORTER2* (*SUC2*) promoter was used to drive expression of an inverted repeat to target the *FD* gene, an exclusively SAM-localized floral regulator. Second, the *SUC2* promoter was used to express an artificial microRNA (*aMiR*) designed to target a synthetic *CLAVATA3* (*CLV3*) transgene in SAM stem cells. Both phloem silencing signals phenocopied the loss of function of their targets and altered target gene expression suggesting that a phloem-to-SAM silencing communication axis exists, connecting distal regions of the plant to SAM stem cells.

**Conclusions:**

Demonstration of phloem-to-SAM silencing reveals a regulatory link between somatic sRNA expressed in distal regions of the plant and the growing shoot. Since the SAM stem cells ultimately produce the gametes, we discuss the intriguing possibility that phloem-to-SAM sRNA trafficking could allow transient somatic sRNA expression to manifest stable, transgenerational epigenetic changes.

**Supplementary Information:**

The online version contains supplementary material available at 10.1186/s12870-022-03998-8.

## Background

Shoots of flowering plants develop from a small population of stem cells located within the shoot apical meristem (SAM). This niche of stem cells is established during embryogenesis and maintained during somatic growth to provide progenitor cells that differentiate into all above-ground tissues, including the flowers that generate male and female gametophytes. In *Arabidopsis thaliana* the stem cell population in the SAM is maintained partly by a feedback loop involving *CLAVATA3* (*CLV3*) [1, 2]. Stem cells at the top of the central zone express *CLV3*, which encodes a non-autonomous signal peptide that moves to subtending cells to inhibit meristem proliferation [3, 4]. Loss of *CLV3* activity results in more cells produced in the SAM, giving rise to larger and additional floral organs [1]. *CLV3* expression is balanced by an endogenous compensation loop [2, 5]. In *clv3* mutants that produce non-functional transcript, this compensation results in a dramatic upregulation of *CLV3* expression [5]. Due to the CLV3 signaling compensation loop, a strong reduction in *CLV3* expression, below 33% of wildtype (Wt) levels, is needed to manifest a ‘clv’ mutant phenotype [6]. For example, in a previous study, only 4/35 inducible *35S* RNA interference lines produced sufficient *CLV3* knockdown to elicit ‘clv’ phenotypes [7]. The CLV3 regulatory circuit is conserved in flowering plants, and subtle variations in activity alter the size and shape of the shoot organs, contributing to the extensive diversity of flowering plants, including crops [8].

Key gene expression changes at the SAM herald shoot developmental transitions. One critical developmental transition at the SAM is the switch from vegetative to reproductive growth, which affects fitness in natural environments and agronomic yield. The initiation of SAM reproductive development is influenced by the environment; for example, long-day (LD) photoperiods hasten flowering time in Arabidopsis [9]. The *FD* gene encodes a bZIP transcription factor that is an important regulator of Arabidopsis flowering. *FD* is constitutively and exclusively expressed in the SAM, and *fd* mutants exhibit delayed flowering under LDs [10, 11]. To trigger flowering, the FD transcription factor interacts with mobile floral inductive signals that are produced in leaf companion cells and transmitted to the SAM via the phloem. Leaves perceive long-day photoperiods and subsequently express *FLOWERING LOCUS T* (*FT*), a phloem mobile signal (florigen) that promotes flowering through interactions with FD [10–12]. The phloem also traffics other information molecules throughout the plant to integrate development with the environment [13, 14]. In addition to protein signals, the phloem transports small RNAs (sRNAs) that can act as gene regulators [15–22].

sRNAs are known to mediate gene silencing in diverse eukaryotes. Plant sRNAs are the products of DICER-LIKE (DCL) enzymatic processing of diverse double stranded RNA (dsRNA) substrates, producing ~ 20–24 nucleotide (nt) sRNA duplexes [23]. *HUA ENHANCER 1* (*HEN1*) then methylates the 3′ end of sRNA to promote RNA stability with *hen1* mutants under-expressing sRNA of all sizes [24]. Mature sRNAs interact with ARGONAUTE (AGO) proteins to repress complementary mRNA through post-transcriptional gene silencing (PTGS) or by repressing transcription through transcriptional gene silencing (TGS) [25]. PTGS typically involves 21/22 nt sRNAs that target complementary mRNA molecules for cleavage or translational inhibition [26–28]. TGS at target genes has been associated with transgenerationally heritable DNA methylation changes that are triggered by the RNA directed DNA methylation (RdDM) cycle [29]. Although not fully understood, non-canonical RdDM links PTGS with TGS; that is, silencing can progress from repressing translation to preventing transcription [30].

MicroRNAs (miRNAs) of 21 nt in length serve as evolutionarily conserved master regulators of many processes, including leaf development, aging, the floral transition and environmental sensing [31–34]. miRNAs are produced from transcripts encoding imperfectly complementary hairpin loops and typically elicit PTGS [23]. However, miRNAs also can trigger TGS either directly [35], or through eliciting secondary sRNA biogenesis [36]. Secondary sRNA synthesis, known as transitivity, often involves RNA-DEPENDENT RNA POLYMERASE6 (RDR6) catalyzed synthesis of dsRNA from a sRNA cleavage product [37, 38], and it is these secondary sRNA that initiate RdDM [36, 39]. Small interfering RNA (siRNA) typically originate from fully complementary dsRNA such as large inverted repeats [23]. In contrast to miRNA, siRNA embody a collection of sRNAs of diverse sizes and regulatory actions.

Some plant sRNAs have been shown to regulate genes non-autonomously by moving short distances from cell to cell through plasmodesmata [40–45]. This local diffusion facilitates the morphogen-like behavior of several miRNAs by creating developmental gradients [32]. In this scenario, miRNA is loaded onto AGO1 in the nucleus or cytoplasm, and AGO1 loading restricts miRNA cell-to-cell mobility [20, 46, 47]. Although not fully understood, cytoplasmic AGO1 miRNA loading, and thus miRNA cellular retention, is antagonized by microtubule-mediated processes [47]. Although the mechanism remains unclear, not all plasmodesmata transmit sRNA equally, as impaired sRNA movement has been observed between the phloem and surrounding tissues as well as regions both within and subtending the SAM [48, 49]. However, less restricted miRNA movement within the SAM and extensive movement in root meristems has also been observed [50, 51]. Mobile sRNA can also elicit transitivity in recipient cells [48, 52–55]. These secondary siRNAs move also, initiate transitivity, and silence targets at a short distance from the initial signal. In this way, secondary siRNAs can establish organism-wide systemic gene silencing by cycling between local sRNA movement and sRNA signal amplification [48, 56, 57]. Additionally, plant sRNA can move long distances via phloem sieve tubes without requiring continual RDR amplification [18, 19]. Phloem-localized sRNA is composed of all size classes, including siRNA and miRNAs that respond to environmental conditions [15, 16]. Long-distance phloem movement occurs across graft junctions [19, 58, 59] and can move into recipient tissues such as root meristems [43] and fully vascularized flowers [58]. Graft mobile sRNA can initiate RdDM, and graft induced alterations in RdDM-dependent methylation has been suggested to increase plant vigor heritably over several generations [17, 60]. However, the extent of phloem-derived sRNA movement into the SAM remains ambiguous [41, 61].

To study the potential movement of long-distance sRNA signals, parallel transgenic approaches were used here to determine whether Arabidopsis sRNA can move phloem-to-SAM to regulate gene expression. In one transgenic system, phloem expression of an inverted repeat targeted the *FD* flowering time regulatory gene that is constitutively and exclusively expressed throughout the SAM. This analysis demonstrated that sRNA derived from a phloem-expressed inverted repeat can move to the SAM and inhibit *FD* activity, resulting in delayed flowering. In a second system aimed at targeting the small population of stem cells in the SAM, a phloem-expressed artificial miRNA (aMiR) was able to repress a synthetic *CLV3* target expressed in these distal cells. This analysis provides additional evidence that silencing signals can move phloem-to-SAM and, specifically, that they can enter an isolated subdomain at the meristem tip. This phloem-to-SAM silencing suggests that somatic sRNA can influence developmental decisions implemented in the SAM. Moreover, since the SAM gives rise to future gametophytes, phloem-to-SAM trafficking of somatic sRNA might trigger epigenetic changes that have transgenerational consequences.

## Results

### Phloem-expressed sRNA can act as an ‘anti-florigen’

**Fig. 1:**
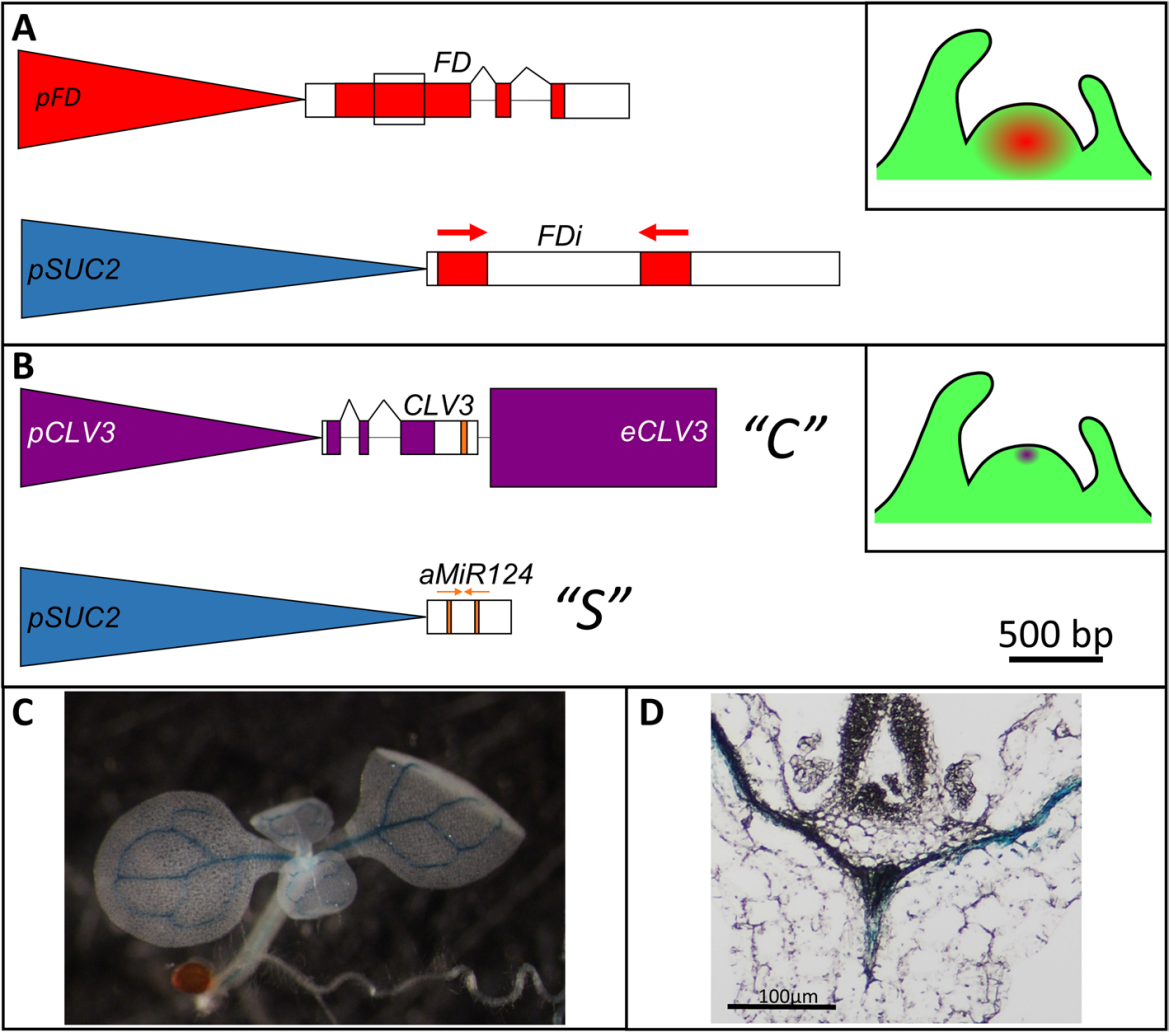
The two Arabidopsis synthetic systems devised to test phloem-to-SAM sRNA silencing. **A** The first system uses a transgenic sRNA source and an endogenous SAM target. The transgene has the *SUC2* promoter (*pSUC2*; blue) driving phloem expression of an inverted repeat (*FDi*) homologous to the native *FD* genomic locus (red). If the phloem-expressed *pSUC2::FDi* can repress endogenous *FD* in the SAM, then the floral transition should be delayed under long day photoperiods. Top Inset: Depiction of where native *FD* is expressed in the SAM. The region in the first *FD* exon homologous to the *FDi* cassette is denoted by a black box. **B** The second system uses a transgenic sRNA source and SAM target. A site complementary to *MmuMiR124* (gold) was inserted into the 3′ UTR of the *CLV3* (purple) transcript. This modified *CLV3* transcript was cloned with the native *CLV3* promoter (*pCLV3*) and 3′ enhancer (*eCLV3*). This *CLV3* transgene (*pCLV3::CLV3: MmuMiR124’::eCLV3*; abbreviated to “*C*”) was then used to rescue *clv3–2* mutants. After selecting for stable homozygous *C* lines, a second transgene was stacked into these plants. This transgene used *pSUC2* to expresses an *aMiR* consisting of the mature *MmuMiR124* sequence in the Arabidopsis *MiR319a* precursor transcript (See Fig. S5 for aMiR secondary structure and the base pairing between *S* and *C* genes). If the *pSUC2::aMmuMiR124* transgene (abbreviated to “*S*”) can silence *C* expression, then the stacked transgenics should show enlarged SAMs and an increase in floral organ numbers. Middle Inset: Depiction of where *CLV3* is expressed in the SAM. **C **&** D** A *pSUC2::GUS:GFP* transcriptional reporter was used to confirm *pSUC2* vein specificity in Col. Both cleared whole mounts (C) and paraffin wax embedded and sectioned tissues (D) exhibited GUS reporter staining constricted to the veins, distal to the shoot apex. The black scale bar denotes the length corresponding to 500 base pairs (bp) of DNA

To determine whether phloem-derived silencing can influence gene regulation in the SAM we utilized two components: a phloem-expressed sRNA signal and a SAM specific target. In Arabidopsis, the well characterized promoter of the *SUCROSE SYMPORTER 2* gene (*pSUC2*) drives specific expression in companion cells, and has been used in previous Arabidopsis phloem-to-SAM movement studies [12, 49, 62–64]. We independently verified the phloem specificity of this promoter by expressing a *pSUC2::β-GLUCURONIDASE:GREEN FLUORESCENT PROTEIN* (*GUS:GFP*) cassette, which exhibited phloem specific reporter activity subtending the SAM in all independent lines (*n* = 3; Fig. 1C, D; Fig. S1). *FD* was selected as the endogenous target, as it is constitutively expressed in the SAM, and loss of *FD* function delays LD flowering which is easily observable [10, 11]. To test phloem-to-SAM sRNA movement, *pSUC2* was used to drive expression of an inverted repeat homologous to *FD* (hereafter *pSUC2::FDi*) (Fig. 1A). As a control to ensure the *FDi* repeat could knockdown *FD* without movement, the *FDi* repeat was cloned under the control of the native *FD* promoter (*pFD::FDi*). We hypothesized that, if sRNA produced by the *pSUC2::FDi* construct can reach the SAM and inhibit *FD* expression, *pSUC2::FDi* transgenics should flower later under LDs.

**Fig. 2:**
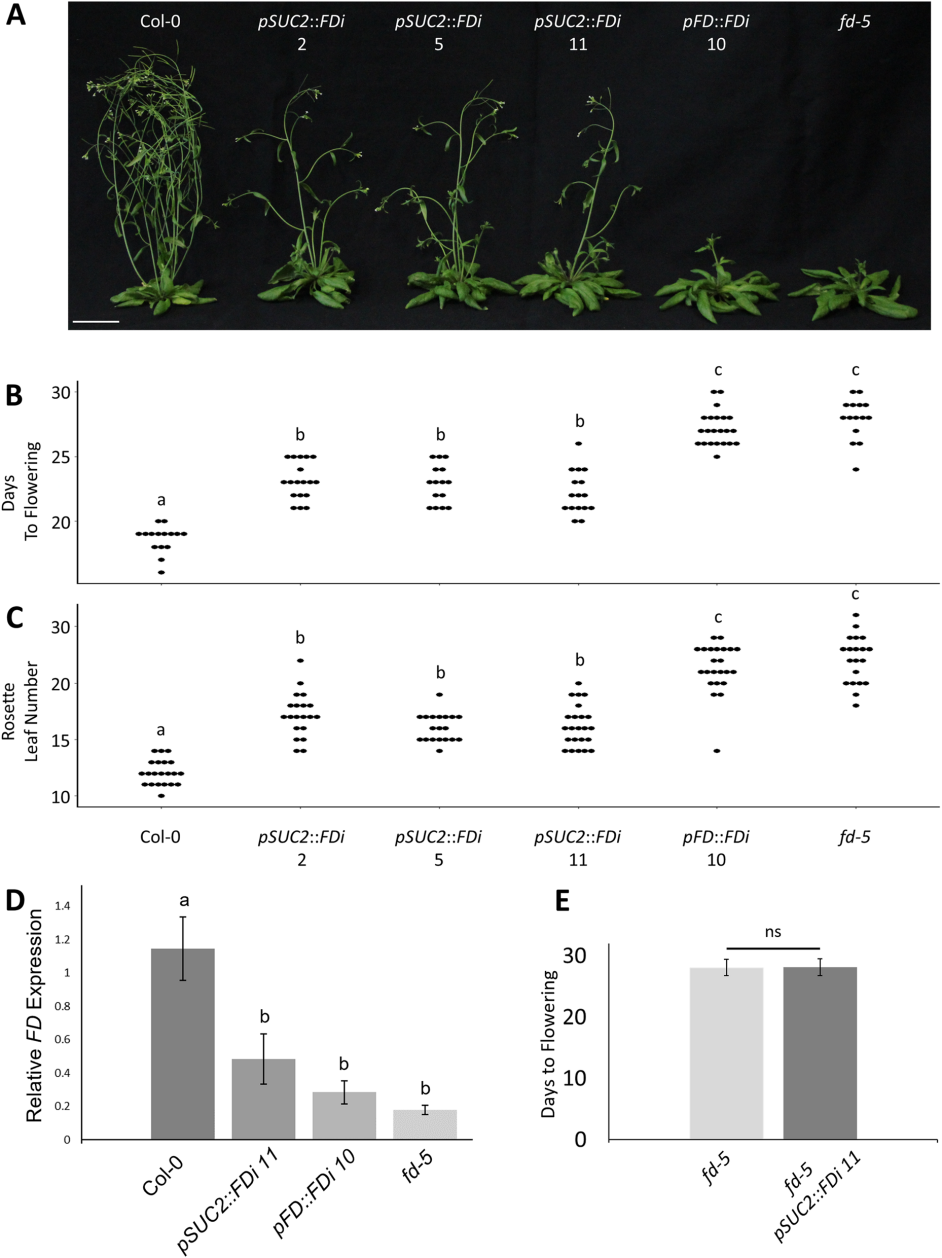
The *pSUC2::FDi* transgene delays the floral transition under long day photoperiods. (A) Plants containing the *pSUC2::FDi* transgene flower significantly later (B) and produce more rosette leaves (C) than the Col-0 Wt control. The *pSUC2::FDi* floral delay was not as strong as that observed by expressing the same *FDi* cassette under the *FD* promoter nor the *fd-5* loss of function mutant. (D) qPCR of apically enriched tissues indicated that the *pSUC2::FDi* transgene reduced *FD* expression to ~ 50% that of Col-0 Wt levels. For each background, qPCR was performed at least in triplicate. (E) *pSUC2::FDi* has no additive effect on flowering when in a *fd-5* background under long day photoperiods. Both *fd-5* and *fd-5* with *pSUC2::FDi* had equivalent flowering times (ns; *p* = 0.85; t-test). Three independent *pSUC2::FDi* lines are shown for all flowering measurements. One representative line was chosen for qPCR and crosses with *fd-5*. Each dot represents one individual for each respective genotype. Plants were photographed 32 days after sowing. Error bars represent standard error (D) and standard deviation (E). Letters denote statistically similar (*p* > 0.05) groups, as determined by ANOVA with post hoc Tukey’s HSD test. To normalize expression to the amount of meristem derived cDNA in the total cDNA pool, *FD* expression was normalized to *KNAT1* expression

Twelve and 17 independent *pSUC2::FDi* lines were created in Landsberg *erecta* (Ler) and Columbia 0 (Col) accessions, respectively. Additionally, 5 and 17 independent lines of the *pFD::FDi* control were created in Ler and Col, respectively. All T2 lines screened displayed variable levels of delayed flowering (Fig. S2). Three Col *pSUC2::FDi* plants with a pronounced delay were selected for further analysis. In progressive generations (up to T7), individuals of these three lines exhibited delayed flowering under LDs that was inherited with the transgene as a dominant trait (Fig. 2A, B, C). Delayed flowering of *pSUC2::FDi* lines was not as strong as that produced by *pFD::FDi* lines nor the *fd-5* mutants (Fig. 2A, B, C). *pSUC2::FDi*, *pFD::FDi* and *fd-5* plants grown under non-inductive short days flowered at the same time as Col Wt (Fig. S3). Quantitative real-time PCR (qPCR) showed that the severity of the floral delay corresponded to *FD* mRNA levels; *pSUC2::FDi* lines exhibited reduced *FD* expression, but the *pFD::FDi* lines and the *fd-5* mutant both had lower *FD* expression (Fig. 2D). Furthermore, crossing the *pSUC2::FDi* transgene into *fd-5* mutant plants revealed no additive delay of flowering, indicating the knockdown was specific to *FD* activity (Fig. 2E). These data indicate that the *pSUC2::FDi* transgene does repress *FD* over a distance and that this distal repression is less effective than proximal knockdown or genetic disruption of *FD*.

### Defects in sRNA production abrogate the *pSUC2::FDi* effect

To provide evidence that the *pSUC2::FDi* floral delay was sRNA driven, a line with a strong floral delay, *pSUC2::FDi 11*, was crossed into mutants that affect sRNA related pathways: *HEN1* [24]*, RDR6* [65–67] and *HASTY (HST)* [65, 68–70]. If the activity of these sRNA related genes is required for *pSUC2::FDi* function, the mutant backgrounds should abrogate the LD floral delay caused by the *pSUC2::FDi* transgene. *pSUC2::FDi hen1–6* plants showed no delay in flowering compared to *hen1–6* alone, suggesting sRNA facilitates knockdown of *FD* (Fig. S4). Similarly, *pSUC2::FDi rdr6–15* plants flowered at the same time as *rdr6–15* mutants (Fig. S4), implicating the involvement of RDR6 amplification in phloem-to-SAM sRNA silencing. The *pSUC2::FDi* gene was also crossed with two different alleles of *HASTY*, an importin/exportin homolog that is implicated in miRNA processing, cell-to-cell export and phloem trafficking [65, 68–70]. Again, the combination of *pSUC2::FDi* with either the *hst-6* or *hst-15* allele suppressed the late flowering phenotype (Fig. S4). However, loss of *HST* function inhibits the actions of floral repressor *miR156,* thus causing an acceleration of the floral transition [71]. Therefore, it is unclear whether the lack of floral delay in the *pSUC2::FDi hst*^*−*^ plants is due to the failure of *FDi* sRNA to act at the SAM or because of aberrant *hst*^*−*^ *miR156* activity. Taken together, these data support phloem-to-SAM mobile sRNA eliciting delayed flowering in *pSUC2::FDi* plants.

### Phloem-expressed artificial miRNA influences gene expression in SAM stem cells

**Fig. 3:**
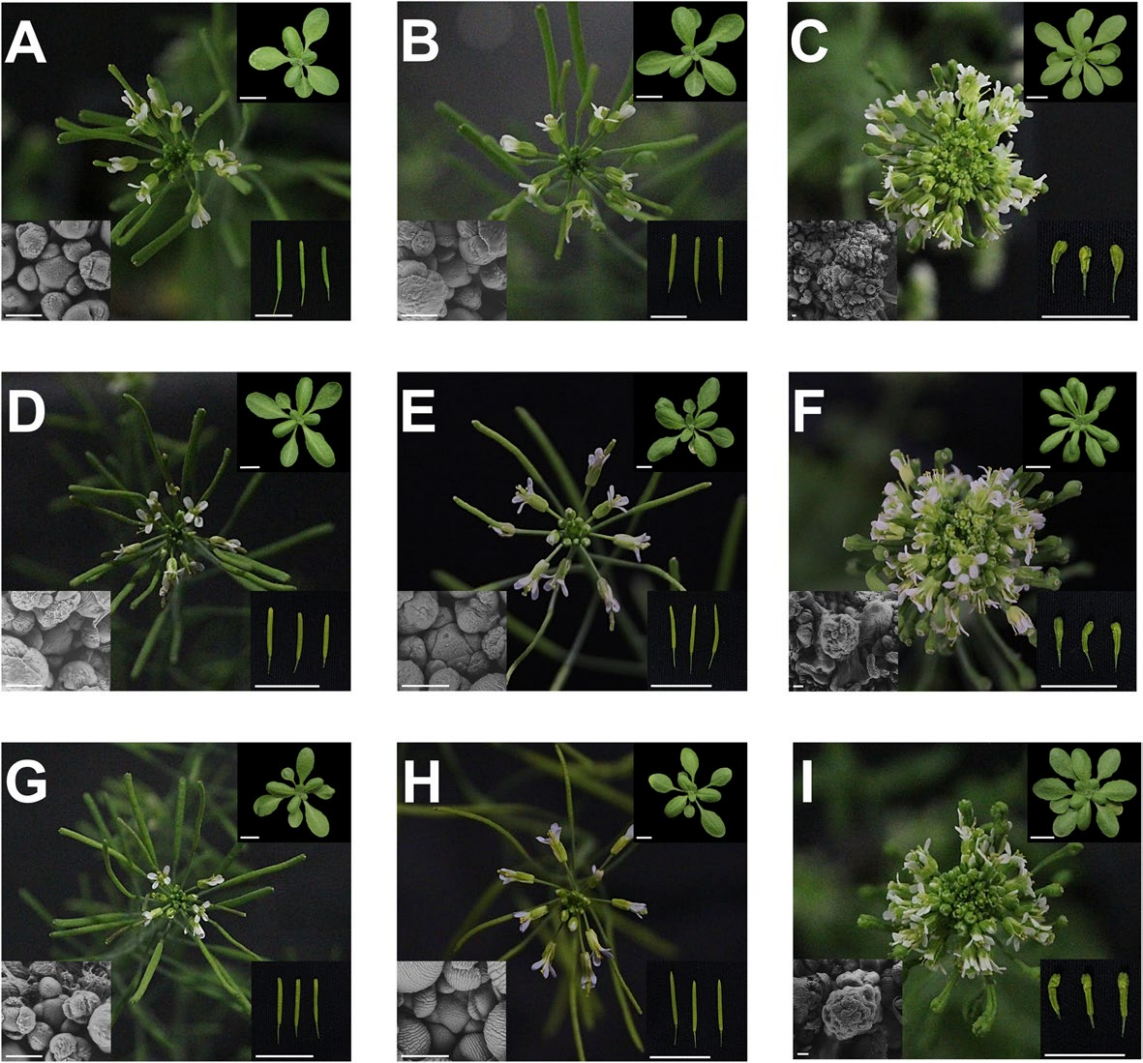
*S- > C* transgenics shows variable silencing with some strong ‘clv’ events. Representative images representing the inflorescence (center), rosette leaves (upper right; scale = 1 cm), silique (lower right; scale = 1 cm) and SAM (bottom left scale = 100 μm) phenotypes for the following groups: (A) Ler Wt (B) Ler *pSUC2::aMiR124* (C) *clv3–2* (D) *C9* (E) *S- > C 9–3* ‘Wt’ (F) *S- > C 9–3* ‘clv’, (G) *C10*, (H) *S- > C 10–5* ‘Wt’, (I) *S- > C 10–5* ‘clv.’ Plants were photographed at 40 days after planting. Note the difference in silique and SAM scale between *clv3–2*, *S- > C 9–3* ‘clv’ and *S- > C 10–5* ‘clv’ groups

To further investigate the movement of sRNA into the SAM, we constructed a second transgenic system to determine whether phloem-derived silencing can affect the SAM and specifically target the activity of *CLV3*, a gene expressed exclusively in the small population of stem cells in the apical region of the meristem [2, 72]. *CLV3* is part of the large *CLE* gene family, members of which are expressed in most Arabidopsis tissues [73]. To avoid off target silencing, which could induce transitivity and silence endogenous *CLV3* without phloem-to-SAM sRNA movement, we designed a system to silence a modified *CLV3* transgene that rescued a *clv3* null background instead of the endogenous *CLV3* gene directly. Our *CLV3* target transgene consisted of the wild-type *CLV3* gene cloned with its native promoter (*pCLV3*) and the 3′ enhancer element (*eCLV3*) in the 3’UTR, which has been demonstrated to cause SAM stem cell expression [49, 72]. However, the 3′ UTR of this *CLV3* transgene was modified to contain a site complementary to mouse microRNA *MmuMiR124. MmuMiR124* was chosen as it has no plant homolog and no complementary sequences were detected in the Arabidopsis genome [74, 75]. This recombinant construct, *pCLV3::CLV3:MmuMiR124’::eCLV3*, is hereafter abbreviated to ‘*C’* (Fig. 1B). The sRNA generating component of this system is an *aMiR* consisting of the Arabidopsis *MiR319a* precursor transcript with the active miRNA site replaced with the *MmuMiR124* sequence expressed under *pSUC2*. This construct, *pSUC2::aMmuMiR124*, is hereafter abbreviated to ‘*S’* (Fig. 1B; Fig. S5). We hypothesized that, if sRNA produced from *S* in the phloem can reach the mRNA expressed from *C* in the stem cells, it should repress *C* expression (*S- > C* silencing) and, consequently, produce a similar phenotype to that exhibited by *clv3–2* mutants. In addition to the previously reported increase in flower and floral organs, we observed that *clv3–2* plants produce additional rosette leaves (Fig. 3C; Fig. S6).

The *C* transgene was transformed into *clv3–2* homozygous mutant plants in the Ler background to ensure that this construct produced functional CLV3 protein and that the *MmuMiR124’* insertion did not affect gene function (Fig. 3A, C). Fifteen independent lines with the *C* transgene all rescued the *clv3–2* phenotype. Likewise, eight independent *C* transgenic lines lacking the *MmuMiR124’* site all rescued the *clv3–2* mutant in the T1 generation. Most rescued plants produced phenotypically ‘Wt’ flowers, except for a few of the first flowers, which produced an occasional extra petal. From these 15 *clv3–2 C* plants, two independent T4 homozygous lines (*C9*, *C10*) were selected for introduction of the *S* gene (Fig. 3D, G). *C9* and *C10* were chosen as they showed the most stable ‘Wt’ phenotypes out of the 15 *C* rescue lines, indicative of stable expression from the t-DNA insertion. Importantly, the *C9* and *C10* lines displayed a stable ‘Wt’ phenotype over many generations (>T5) and individuals (> 1000); i.e., in these genotypes no ‘clv’ phenotypes were ever observed.

The *S* gene was then transformed into the *C9* and *C10* lines (see Fig. S7 for a pedigree of the experiment) and 28 and 27 independent *S* lines were recovered in the *C9* and *C10* backgrounds, respectively (hereafter *S- > C 9* or *S- > C 10*). In the T1 generation, other than some minor aberrations in floral bud formation, all lines appeared phenotypically ‘Wt’ (Fig. S8). In the T2 generation, 27 *S- > C* lines displayed minor qualitative changes in inflorescence buds, including slight changes in petal number and position, but otherwise appeared to be ‘Wt’ (Fig. S9). However, three T2 families, *S- > C 9–2*, *S- > C 9–3,* and *S- > C 10–5* produced phenotypically ‘clv’ plants (Fig. 3; Fig.  S9). One other line, *S- > C 9–11*, produced enlarged flowers with more and larger petals and sepals. However, since this line did not phenocopy the prototypical *clv3* loss of function, it is not discussed here further. Once the ‘clv’ phenotype appeared in the *S- > C* lines it was inherited in all self-cross progeny. In all *S- > C* lines that contained ‘clv’ individuals, some ‘Wt’ individuals would produce ‘clv’ and ‘Wt’ progeny. Quantifying this segregation ratio from *S- > C 9–3* ‘Wt’ plants homozygous for *S*, revealed the production of ‘clv’ progeny at a rate near 25% (102/350 T5 plants; *p* = 0.07; Χ^2^ test vs 0.25), suggesting the ‘clv’ phenotype was controlled by one recessive locus (Fig. 3E, F).

**Fig. 4:**
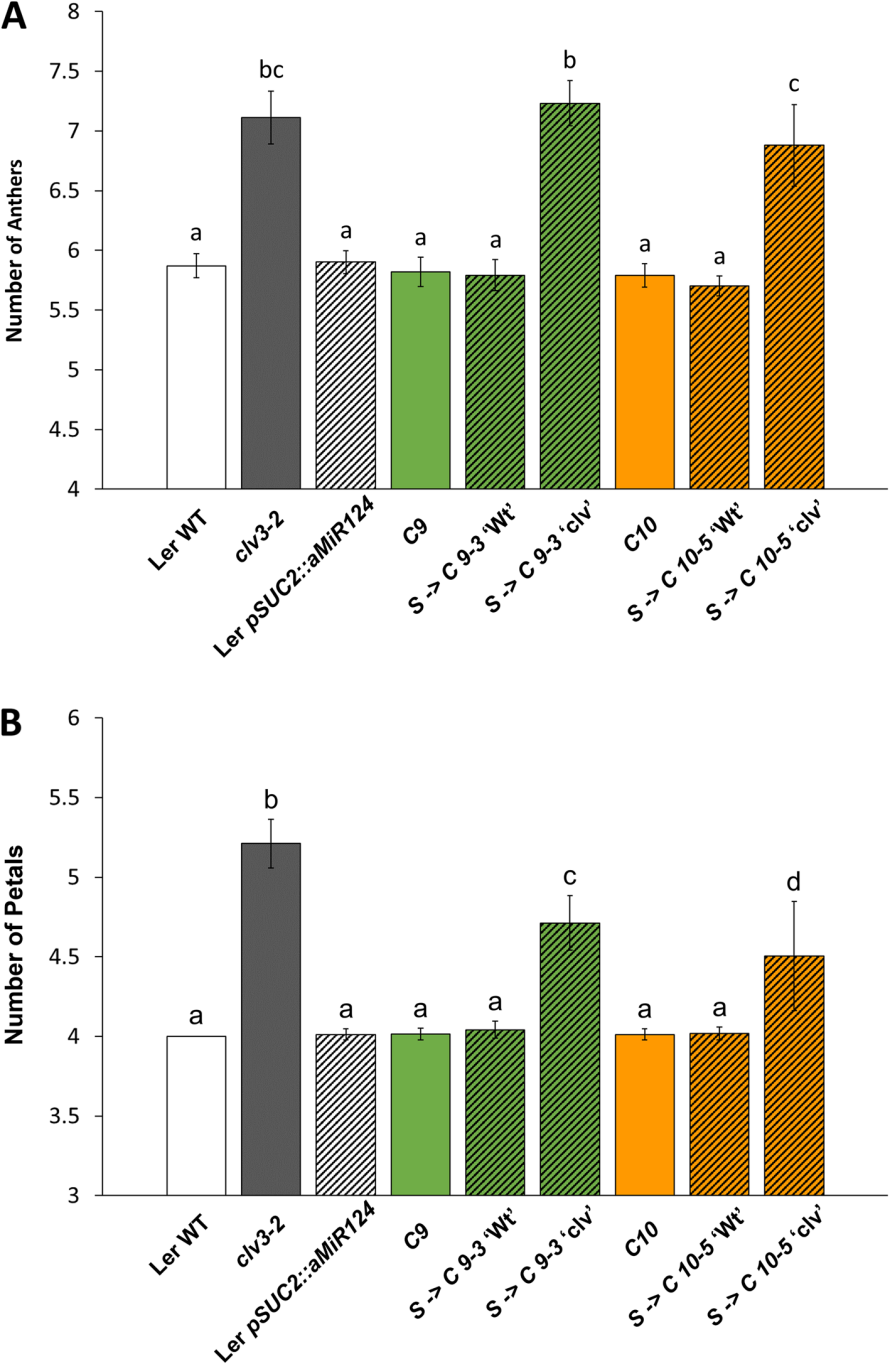
The two independent *S- > C* ‘clv’ lines produce additional anthers and petals per flower. All ‘clv’ groups produced aberrant numbers compared to Ler Wt, the *C* only lines, or the *S- > C* ‘Wt’ groups. **A** Both *S- > C 9–3* ‘clv’ and *S- > C 10–5* ‘clv’ lines produced a similar number of anthers per flower compared to *clv3–2*. **B** However, *clv3–2* produced more petals per flower than either line. Letters denote statistically similar (p > 0.05) groups. For anther counts *p* values were determined ANOVA with post hoc Tukey’s HSD test. For petal counts, due to the zero variance in Ler Wt, p values were determined by a Kruskal-Wallis rank sum test followed by pairwise Wilcox tests. Error bars indicate +/− standard error. For each group, floral organ counts were conducted on 6 or more individuals

The *S- > C* ‘clv’ plants exhibited a qualitatively less severe phenotype than the *clv3–2* mutant, with visibly less undifferentiated tissue in the inflorescence meristem and less severe silique phenotypes (Fig. 3). Additionally, *S- > C* ‘clv’ plants often produced flowers with tightly closed sepals, whereas this was rarely observed in the *clv3–2* mutant. To quantify the severity of the ‘clv’ phenotype in the *S- > C 9–3* and *S- > C 10–5* lines, the number of anthers and petals produced per flower were determined (Fig. 4). Both *S- > C 9–3* and *S- > C 10–5* plants displayed numbers of anthers typical of the *clv3–2* mutant. However, *clv3–2* plants produced more petals than either *S- > C* ‘clv’ group. Therefore, both *S- > C* ‘clv’ lines exhibit phenotypes consistent with loss of *clv3* function, but not as severe as the *clv3–2* null.

To ensure that the *S* transgene itself cannot cause a phenotype resembling loss of *clv3* function, *S* alone was transformed into the Ler Wt background (Fig. 3B). Of 9 independent *S* lines, none exhibited a *clv*-like phenotype in generations T1 to T4. Likewise, crossing *S* into the *clv3–2* mutant background had no effect on ‘clv’ phenotypes. A high proportion *S* lines produced large, serrated rosette leaves, which was also seen in the *S- > C* lines including the three lines that produced ‘clv’ individuals (Fig. S10). Additionally, these *S* plants had a delayed floral transition, producing more rosette leaves than Ler Wt (Fig. S11). Like most transgenic phenotypes that vary with expression levels, these *S* induced phenotypes ranged in severity between independent lines, with the strongest phenotypes appearing in the lines that exhibited *C* silencing. These strong *S* induced phenotypes likely reflect high *aMiR* expression levels and, since the strong *S* phenotypes were present in all independent *S- > C* lines producing ‘clv’ individuals, we suspect that high *aMiR* expression is a prerequisite for *C* silencing. The increase in rosette leaf number in *S* plants appeared additive with the ‘clv’ mediated increase in rosette leaf number, with *S- > C* ‘clv’ plants producing more rosette leaves than *clv3–2* plants (Fig. S12). These observations suggest that, despite the lack of genomic homology, phloem expression of *MmuMiR124* affects Arabidopsis development, but *S* alone does not cause ‘*clv’* phenotypes.

**Fig. 5:**
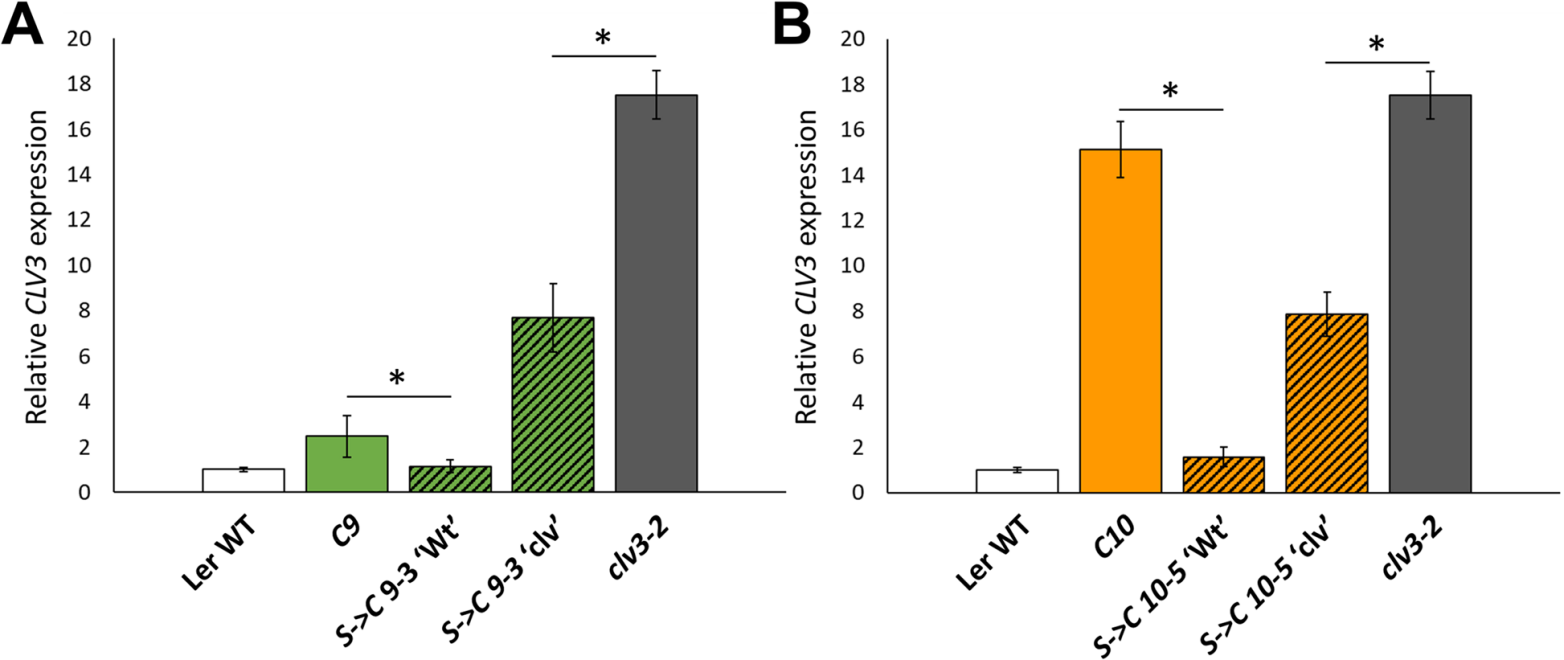
qPCR of apically enriched tissues showed differing *CLV3* expression in *S- > C* ‘Wt’ and *S- > C* ‘clv’ plants. For two independent events, (A) *S- > C 9–3* and (B) *S- > C 10–5*, *CLV3* expression was reduced in *S- > C* ‘Wt’ plants compared to that observed in their respective *C* only progenitors. Both *S- > C 9–3* ‘clv’ and *S- > C 10–5* ‘clv’ groups show elevated *CLV3* expression. However, this expression was not as high as that found in the *clv3–2* background. Displayed p values were determined by a student’s t-test. Error bars indicate +/− standard error. For each background, qPCR was performed in at least triplicate

In *S- > C* lines that produced both ‘Wt’ and ‘clv’ plants, quantitative real-time PCR (qPCR) was used to measure total *CLV3* transcript levels; that is, the sum of expression from the both non-functional *clv3–2* locus and *C*. Both *S- > C 9–3* ‘Wt’ and *S- > C 10–5* ‘Wt’ plants showed a decrease in *CLV3* expression compared to the *C9* and *C10* lines, respectively (Fig. 5). This decrease in *CLV3* expression suggests that *S* reduces *C* expression in *S- > C* ‘Wt’ plants, but not enough to manifest a ‘clv’ phenotype. The *clv3–2* plants used in this study produced a large amount of transcript, similar to what was reported for the *clv3–9* null allele [1, 5]. *S- > C 9–3* ‘clv’ and *S- > C 10–5* ‘clv’ plants also demonstrated an increase in defective *CLV3* transcript (Fig. 5). However, consistent with the milder ‘clv’ phenotypes in the *S- > C* lines, both *S- > C* ‘clv’ lines had reduced *CLV3* expression compared to the *clv3–2* background, suggesting low levels of transgenic *CLV3* transcript and activity may remain in the *S- > C* lines. These data show that mobile *S* derived sRNA may decrease *C* expression and, in the *S- > C* events that do trigger a ‘clv’ phenotype, the plants over-express non-functional *clv3–2* transcript.

### Strong *S- > C* ‘clv’ silencing events do not require continued *S* presence and show transgenerational shifts in ‘clv’ phenotypic severity

Since *S- > C* ‘clv’ events are consistent with the inheritance of a single recessive locus, we investigated whether the *S* gene was no longer required for the ‘clv’ phenotype. That is, is the appearance of the ‘clv’ phenotype in the T2 generation due to permanent silencing of the *C* allele in the T0/T1 generation by high levels of *S* expression? To check this, a *S- > C 9–3* ‘clv’ plant was crossed to the homozygous *C9* parental line. If the ‘clv’ phenotype requires continuous *aMiR* silencing, the F2 plants from this cross, which have at least one copy of *S* and two copies of an unlinked recessive *C* allele, 3/16th of the F2 plants should be ‘clv’. However, if ‘clv’ is caused by one recessive allele alone, ¼ of the *C9* x *S- > C 9–3* ‘clv’ F2 population should be ‘clv’. All F1 plants were ‘Wt’ and 152/528 F2 plants exhibited a ‘clv’ phenotype. This is not consistent with a model that requires the continuous interaction between *S* and *C* (Χ^2^ test vs 3/16; *p* = 3.433e-9). The observed ratio is close to that expected under the single locus model (Χ^2^ test vs ¼; *p* = 0.0465), consistent with the ‘clv’ phenotype no longer requiring the presence of *S.* However, as the number of ‘clv’ plants did not exactly adhere to the predicted single locus Mendelian ratio, it is possible the *C* phenotype is not always inherited faithfully. Furthermore, several *C9* x *S- > C 9–3* F2 ‘clv’ plants were genotyped for the presence of the *aMiR* cassette. Of these ‘clv’ plants, 14/64 (Χ^2^ test vs ¼; *p* = 0.56) lacked *S*. The ‘clv’ phenotype was maintained in the absence of *S* for at least two generations. Progeny from four of the F2 lines that did not have the *aMiR* cassette (which contains a kanamycin resistance gene) were grown on selection and all families lacking *S* exhibited kanamycin sensitivity. This again suggests that *S* assorts independently of the ‘clv’ trait. Therefore, *S* likely acted in the T0/T1 generation to silence 1 *C* allele but is not required to maintain the ‘clv’ phenotype.

**Fig. 6:**
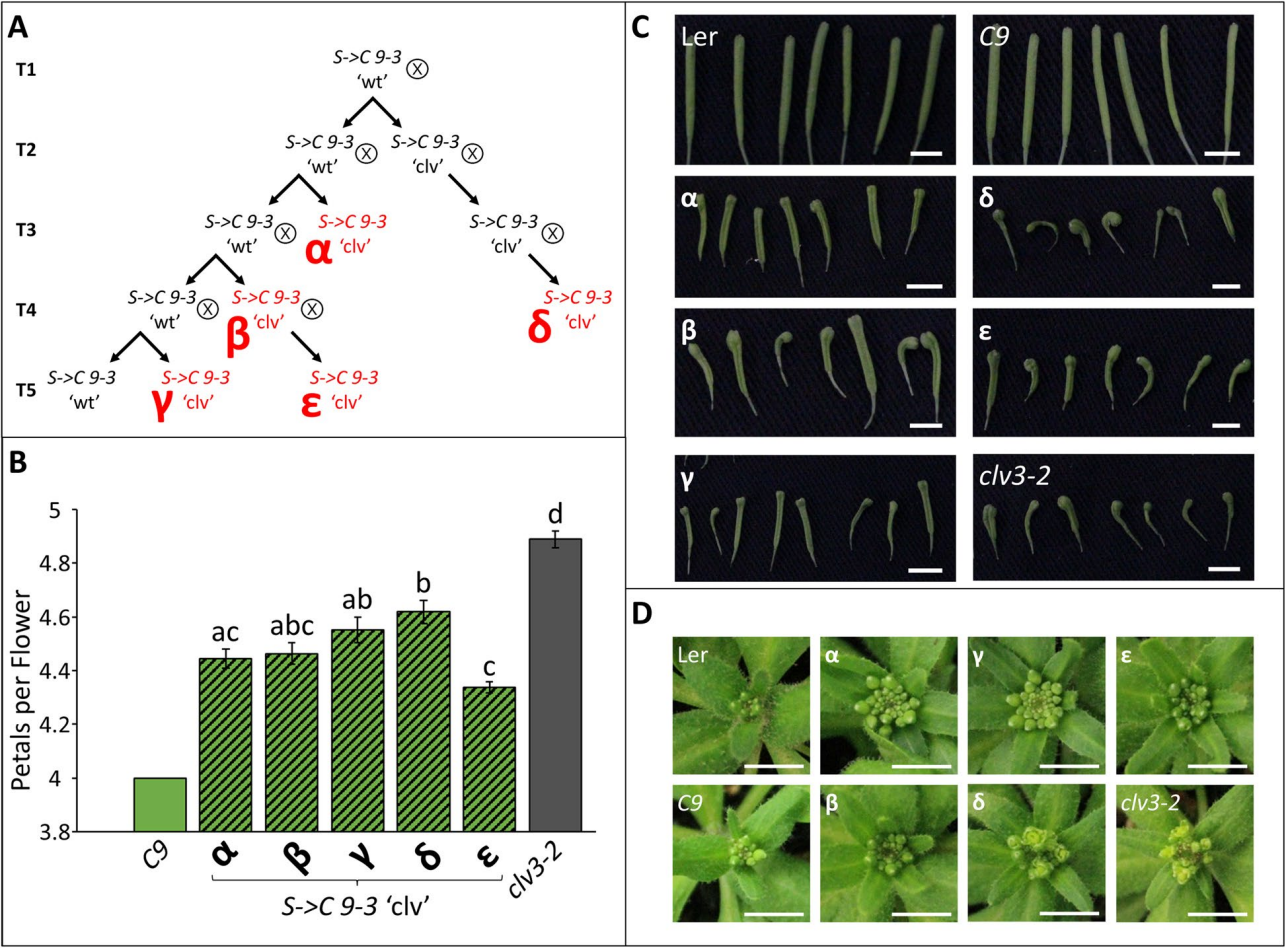
*S- > C* ‘clv’ families exhibit phenotypic variation between genetically identical plants. **A** A pedigree outlining the relatedness of the sampled *S- > C 9–3* families. Sampled families are written in red and identified by a Greek letter (α-ε). All families were homozygous for both *S* and *C*. **B** The number of petals produced per flower varied within different *S- > C 9–3* ‘clv’ families.’ **C** Silique and **D** developing inflorescence phenotypes produced by the *S- > C 9–3* transgenic families. Mirroring the petal number data, with the δ family exhibiting the most severe qualitative ‘clv’ phenotypes. Letters denote statistically similar (p > 0.05) groups, as determined by ANOVA with post hoc Tukey’s HSD test. *C9* was excluded from this statistical analysis to allow the use of a parametric test. Error bars indicate +/− standard error. Scale bars denote 0.5 cm. For each group in B, petals were counted for at least 11 individuals

A possible explanation for the low frequency of *S- > C* ‘clv’ events, and their eventual independence from *S*, is that *S* elicited stable epigenetic silencing at *C* in the T0/T1 generation. Infrequent appearance of strong *C* silencing resembles the rarity of endogenous epiallele formation in Arabidopsis [76, 77]. Less likely, an unknown mechanism could cause a T0/T1 genetic change producing the *S- > C* ‘clv’ phenotype. If such a change caused the *S- > C* ‘clv’ phenotype, all plants within the same transgenic line should display an identical ‘clv’ effect. Inconsistent with this genetic explanation, within the same independent line the severity of the ‘clv’ phenotype changed significantly in subsequent generations (Fig. 6). An independent growth experiment comparing 2 *S- > C 9–3* T4 families again supported ‘clv’ phenotype differences within the same transgenic lines (Fig. S13). Overall, this observed variation in phenotype is at odds with a genetic explanation of the *S- > C* ‘clv’ events, supporting the hypothesis that epigenetic changes occurred in the T0/T1 generation.

### *S- > C* phenotypes exhibit somatic instability

**Fig. 7:**
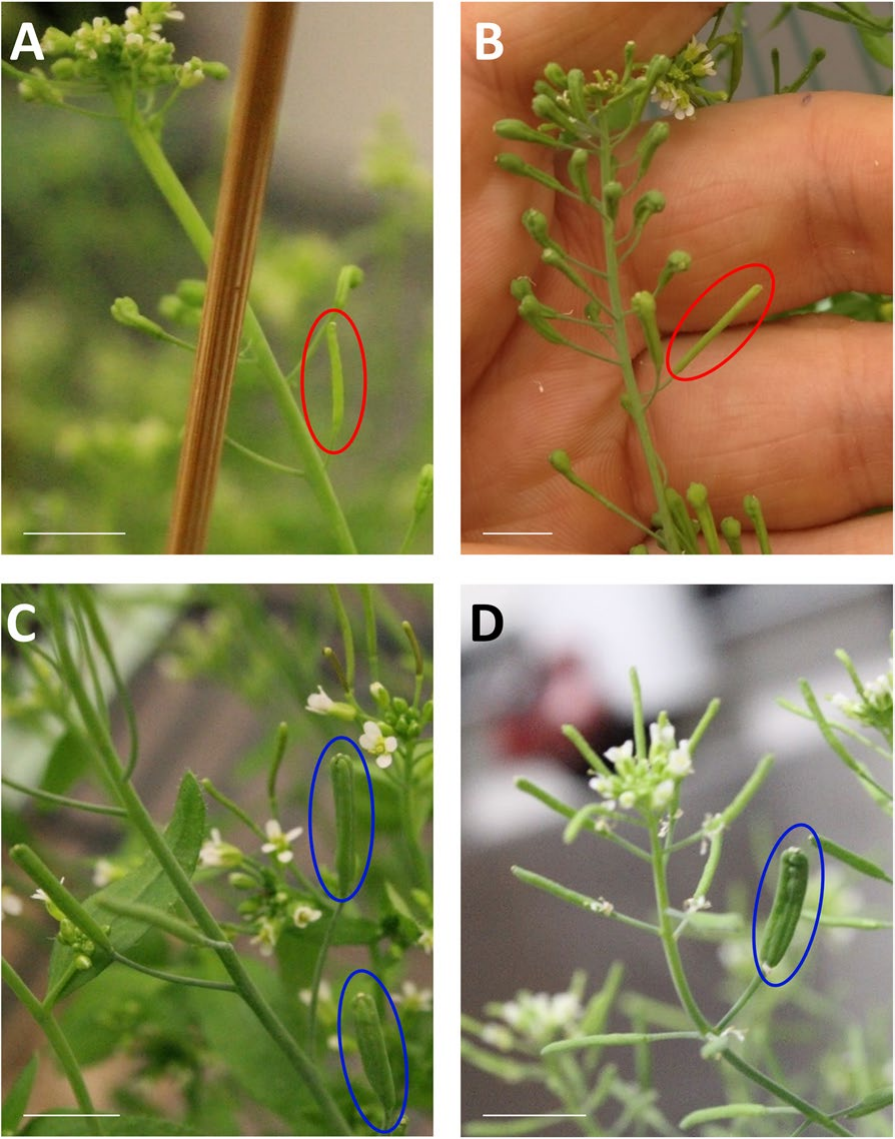
*S- > C* plants exhibit somatically unstable phenotypes, consistent with revertant or silenced sectors. *S- > C* ‘clv’ plants can produce one or two bi-carpellate ‘Wt’ siliques (A&B, red ovals). Likewise, *S- > C* ‘Wt’ plants can produce ‘clv’ multi-carpellate siliques (C&D, blue ovals). Scale bar denotes 1 cm

Close observation of *S- > C* individuals revealed occasional somatic instability, with ‘clv’ plants producing ~ 1–3 siliques that appeared phenotypically ‘Wt’ (Fig. 7; Fig. S14). These apparent reversions occurred in *S- > C* ‘clv’ transgenics as well as *S- > C 9–3* ‘clv’ x *C9* F3 plants that no longer contained *S*. ‘Wt’ siliques were never observed in *clv3–2* mutants (Fig. S14). These unstable phenotypes [78, 79] are consistent with the hypothesis that the *C* silencing is epigenetic in nature. Seed produced from ‘Wt’ siliques (on an otherwise ‘clv’ plant) were sown to check for changes in progeny phenotype*.* Two out of 288 plants from these ‘Wt’ siliques produced phenotypically ‘Wt’ plants, suggesting these somatic sectors are caused by heritable reversions. Conversely, although rarer, *S- > C* ‘Wt’ plants could produce ‘clv’ siliques with 3–4 carpels, suggesting both *C* alleles had been silenced in a sector (Fig. 7). Seed from ‘clv’ siliques on an otherwise ‘Wt’ plant produced ‘Wt’ plants only, indicating the sectored silencing was not heritable. Taken together, these observations reveal somatic instability of both the ‘Wt’ and ‘clv’ phenotypes in the *S- > C* background. To investigate the possibility that the *C* locus in ‘clv’ individuals is methylated, ‘clv’ plants were treated with the DNA methylation inhibitor 5-Azacytidine (5-Aza). Ler Wt plants simultaneously treated with the concentration of 5-Aza used here displayed no developmental aberrations. However, one of 719 F3 from ‘clv’ seeds treated with 5-Aza produced a ‘Wt’ plant (Fig. S15). The progeny of this 5-Aza revertant plant segregated ‘clv’ as a recessive trait, consistent with 1 *C* allele having regained function (Χ^2^ test vs ¼; *p* = 0.84 *n* = 34). The ‘Wt’ progeny of this 5-Aza revertant displayed flowers with unclosed sepals, which was not observed in *C9* or *S- > C 9–3* ‘Wt’ lines but is seen in ‘clv’ individuals. This is consistent with the 5-Aza treatment reactivating 1 *C* allele, although not to the same level as the naïve state before *S* introduction.

### *S- > C* plants display variable shoot morphologies that are not heritable

Unexpectedly, *S- > C* transgenic plants exhibited a significant level of shoot aberrations. Examples include inflorescences that end in terminal flowers or plants that lose apical dominance (Fig. S16). Paradoxically, similar bud termination effects have been observed in plants that overexpress *CLV3* [6]*,* suggesting an imbalance of CLV3 signaling in *S- > C* plants. This phenotypic variability was more pronounced after the plants were outcrossed. In 3 *S- > C* F2 populations, a wide array of unexpected polymorphisms appeared (Fig. S17). None of these phenotypes were inherited in F3 progeny, although the F3 generation likewise produced individuals with a similar range of shoot aberrations. F2 populations from Ler x *S* crosses displayed no phenotypic instability, suggesting the instability required both *S* and *C*. One commonly observed phenotype was inflorescences that terminated with multi-carpellate or multi-staminate structure, reminiscent of flowers in *carpel factor*y (aka *dcl1*) mutants [80]. These variable growth aberrations suggest that interaction of *S* and *C* might stochastically disrupt shoot growth and development, possibly by interfering with endogenous phloem-to-SAM sRNA signaling. The diversity and severity of these phenotypes may hint at undiscovered regulatory roles of endogenous phloem-to-SAM sRNA communication *in planta*.

## Discussion

### Phloem-derived sRNA can influence gene expression in the SAM

Mobility of sRNA within the plant has been established, yet the extent of this movement with respect to phloem-to-SAM remains unexplored [41, 61]. Cell-to-cell systemic transitive silencing can reach the SAM, but this represents the culmination of a series of short distance events, not long-distance trafficking between distant regions of the plant. Importantly, phloem-to-SAM sRNA trafficking allows for distally generated sRNA signals to reach the SAM, even if they cannot induce systemic transitivity, allowing the SAM to be influenced by diverse sRNA signals originating in remote parts of the plant. Although our experiment cannot differentiate between the movement of sRNA or their dsRNA precursors, there is ample evidence that it is sRNA itself that is mobile *in planta* [17, 18, 47, 58, 59, 81], and in the phloem specifically [16, 21, 22]. Therefore, the transgenic systems described here suggest that an axis of communication from phloem-to-SAM via sRNA messages does exist and, further, provides preliminary insights into how endogenous phloem-to-SAM sRNA signaling might occur.

In both synthetic silencing systems described here, the sRNA signal originates in phloem companion cells and migrates to different targets. The *pSUC2::FDi* system demonstrated that fully complementary inverted repeat RNA expressed in phloem could downregulate gene expression in the SAM. Curiously, *FD* repression appeared to not occur in the *rdr6–15* mutant (Fig. S4). RDR6 catalyzes the creation of dsRNA from ssRNA precursors [54], a step that is not needed to produce sRNA from inverted repeats [55, 65], like *FDi*. Therefore, *rdr6–15* must disrupt long distance silencing downstream of the initial sRNA biogenesis. Inverted repeats generate a spectrum of sRNA sizes that can trigger secondary sRNA biogenesis and transitivity, and this is often lost in the *rdr6–15* background [82, 83]. Importantly, *FD* is expressed exclusively in the apex [10, 11]. Therefore, secondary sRNA biosynthesis is not needed for sRNA to reach the SAM, as it could only occur after the sRNA has reached *FD*. Thus, we hypothesize that RDR6-mediated transitivity is a critical step in phloem-to-SAM sRNA signal reception, amplifying a weak mobile signal to elicit gene silencing after it arrives at the target tissue. miRNAs have been reported to trigger secondary sRNA biogenesis [36, 55], so the *S- > C* system could likewise rely on RDR6 actions in the SAM. In this way, only small initial populations of phloem mobile sRNA need to reach the SAM to trigger an effective silencing response.

### Phloem sRNA signals can target stem cells in the shoot apex

The *S- > C* silencing system combined a phloem-expressed *aMiR* with a synthetic *CLV3* target expressed in SAM stem cells. Most *S- > C* transgenics were phenotypically ‘Wt’ but showed reduced *CLV3* transcript levels, supporting phloem-to-SAM trafficking of *S*-derived miRNA to the central zone. Only a few independent lines produced ‘clv’ individuals, which is consistent with the requirement for strong silencing of *CLV3* in order to manifest meristem over-proliferation [6, 7]. Although we cannot conclusively exclude an alternative unknown mechanism, we favor the explanation that epigenetic processes underly the *S- > C* ‘clv’ events, as supported by the appearance of revertant somatic sectors and significant phenotypic variation within the same transgenic line.

Previous study of miRNA intra-SAM movement describes distinct cellular domains wherein miRNAs act non-autonomously; outside of these domains, non-autonomous miRNA silencing was reported to be restricted [49]. In contrast, our *S- > C* system supports miRNA movement from the phloem through these domain barriers to stem cells in the central zone. In most instances (*S- > C* ‘Wt’ plants), *S*-triggered reduction in *CLV3* expression was modest, perhaps below levels that can be detected by the histological approach used by Skopelitis et al., (2018). Additionally, the transgenics described in Skopelitis et al., (2018) were generated in the *rdr6*^*−*^ background, which may preclude strong silencing events in the T0/T1 generation (see below). Taken together, we suggest that sRNA gating within the SAM is not impregnable, such that some amount of miRNA may pass through these domain barriers. Indeed, a similarly leaky sRNA gating mechanism was observed in the parenchyma subtending the SAM [48].

### Stochastic T0/T1 events may allow for rare but strong *C* silencing events

The appearance of *S- > C* ‘clv’ in the T2 generation suggests that only 1 *C* allele was silenced in the T0/T1 generation. Therefore, we speculate that initial *trans*-action of *S* triggered *cis* silencing occurred at 1 *C* allele. These strong silencing events all occurred in *S* lines that exhibited strong *S* induced phenotypes, implying high level of *aMiR* expression. The rarity of such events (3/55 *S* insertions; 3/110 *C* alleles) suggest a stochastic trigger for strong *C* silencing which we suspect is predicated on high *aMiR* expression. The *C aMiR* site is close to the transcriptional terminator, disruption of which has been associated with sRNA-mediated transgene silencing [72, 84]. If *S*-derived sRNA initiates RdDM, it may spread DNA methylation [85, 86] onto the linked terminator sequence, silencing *C*. miRNAs have been reported to elicit RdDM, often by triggering transitivity, and we suspect this entry into RdDM could be the stochastic event behind strong *C* silencing. In *Nicotiana benthamiana*, *miR319a* produces 21 nt miRNAs, which typically are not associated with transitivity [87]. However, the *aMiR* used here has an altered miRNA sequence, and miRNA structure can cause 22 nt miRNA production and transitivity [88]. Therefore, if *S*-derived 22 nt sRNA can reach the central zone it could silence expression from the *C* transgene. Alternatively, both *AGO7* (transitivity-associated) [89, 90] and *AGO6* (RdDM-associated) [91] are expressed in the central zone [92] and could interact with *S*-derived sRNA to silence *C*.

We suggest that all strong *C* silencing events occurred in the T0/T1 generation, as no ‘clv’ events emerged later from transgenic lines that did not show T2 ‘clv’ phenotypes. The T0/T1 generation is critical for sRNA silencing of newly inserted transgenic transposons, with T0/T1 sRNA silencing mutants having transgenerational effects on the efficacy of silencing [93]. Since strong *C* silencing likely occurred shortly after *S* integration, it is possible that integration transiently affects sRNA silencing of not only the newly integrated sequence, but also of any homologous loci. T-DNA transgenes are propagated in bacteria, and thus lack most cytosine methylation and any pre-existing histone state upon integration. Perhaps establishment of a de novo chromatin state during *S* integration enables the plant genome to occasionally facilitate RdDM at *C*. DNA integration involves dsDNA breaks, which triggers double-stranded break-induced small RNA (diRNA) expression and are thought to recruit RNA Pol IV and V [94, 95]. How dsDNA break triggered events affect de novo chromatin modifications remain unclear, but diRNA expression involves *RDR6* [94]*,* and could provide a brief window wherein the *S* transgene is capable of strong *C* silencing. Importantly, diRNA synthesis requires RNA Pol II transcription of the integrated sequence [95], so any *S* diRNA would be co-expressed with *pSUC2* and, if they are needed to silence *C*, still require phloem-to-SAM trafficking. Future investigation into how molecular events associated with integration influence sRNA silencing in *cis* and *trans* will shed light on this process.

### Long distance sRNA trafficking could hypothetically induce epigenetic changes at the SAM

The role of sRNA in mediating gene silencing is well established, and mobile sRNA has been shown to alter DNA methylation in tissues, including the root apical meristem [17, 19, 96]. We suspect similar RdDM events may occur after mobile sRNA enters the SAM. Graft mobile sRNA movement into Arabidopsis flowers, and infrequently into male meiotic tissue, has been demonstrated previously [58], suggesting that distally produced sRNA has the potential to influence the nascent next generation. However, our demonstration of phloem-to-SAM sRNA movement suggests somatic sRNA expressed at any developmental stage prior to flower formation has the potential to influence the next generation. Somatic sRNA could enter the phloem stream and move to stem cells to influence gene expression via RdDM, ultimately affecting future gametes produced from the SAM. It is intriguing to speculate that, since plants do not completely erase DNA methylation during gametogenesis [97], DNA methylation changes caused by phloem-mobile RdDM might be stably inherited for many generations. The composition of sRNA populations in phloem change with the environment [15, 16], raising the possibility that transient environmentally-induced sRNA could have lasting epigenetic consequences in both current and future generations. Indeed, others have suggested that sRNA could bridge environmental sensing with progeny imprinting [61, 98–100]. Our demonstration of phloem-to-SAM sRNA transport provides a route for these adaptive epigenetic changes to occur, and future work should investigate this possibility in wild or agronomic plant populations.

## Conclusions

The transgenics systems developed here suggest that silencing signals from the phloem can influence gene expression in the SAM, and specifically in the central zone stem cells. Phloem expression of an inverted hairpin caused more consistent SAM gene silencing than phloem *aMiR* expression, with the latter causing rare strong silencing events. This demonstration of phloem-to-SAM silencing provides a route for distal somatic sRNA to influence the SAM without eliciting organism-wide systemic gene silencing.

## Methods

### Plant materials and growth conditions

*Arabidopsis thaliana* plants were grown in Sunshine mix LA4 or Promix BX at 60% relative humidity, with an irradiance of 150 μmolm^− 2^ s^− 1^ and day/night temperatures of 22 °C and 18 °C respectively. Plants were fertilized bi-weekly with liquid 17–5-17 (200 ppm) fertilizer. Plants were grown under 16-hour long days photoperiods, or 8-hour short day photoperiods when specified. Experiments were conducted in the Landsberg *erecta* (Ler) or Columbia-0 (Col) accessions. Arabidopsis mutants (*clv3–2*, *fd-5*, *hen1–6*, *hst-6*, *hst-15*, and *rdr6–15*) were obtained from the Arabidopsis Biological Resource Center (ABRC). Hand crosses were conducted using unopened female flowers and dehiscent anthers.

### Recombinant plasmid construction

To construct the *pSUC2::FDi* and *pFD::FDi* transgenes, a 264 bp region of the first exon of *FD* was amplified from cDNA and inserted into *pHANN* in sense and anti-sense orientation. This region was selected to minimize off-targeting to other genes homologous to conserved domains in *FD*. This hairpin-forming fragment was then amplified and inserted into *pDONR221 P5P2* via Gateway® recombination (Invitrogen). The *SUC2* promoter sequence [62] was amplified from a plasmid graciously provided by George Coupland and recombined into *pDONR221 P1P5r*. The *FD* promoter was amplified from an Arabidopsis BAC clone (F4B14 from the ABRC) and likewise inserted into *pDONR221 P1P5r*. A 3-way Gateway® reaction recombined promoters with *FDi* into *pK7WG*.

The *pSUC2::GUS:GFP* reporter construct was created by Gateway® recombination cloning *pSUC2* into *pDONR P4P1r*, then recombining this into *pKGWFS7*. To construct *C*, the native *pCLV3::CLV3::eCLV3* locus was amplified from Col-0 genomic DNA in two fragments and subcloned into *pUC19* through restriction enzyme cloning. Within the 3′ *CLV3::eCLV3* fragment, a naturally occurring NsiI site (New England Biolabs) was used to insert an oligonucleotide duplex containing the *MmuMiR124’* site into the *CLV3* 3′ UTR. Next the two *pCLV3::CLV3::eCLV3* fragments were digested and inserted into *pBM42GW,3* via a three fragment ligation. A rescue control was likewise constructed using 3′ *pCLV3::CLV3::eCLV3* without the *MmuMiR124’* insertion. To construct *S*, *pSUC2* was inserted into *pK7m24GW,3* via restriction enzyme cloning. Gene synthesis (Eurofins-Operon) was used to create the *aMiR* by replacing the active *miRNA319a* site with that of *MmuMiR124*. *MmuMiR124* sequence was modified to have one additional 3′ adenosine, often present in the sequencing reads on miRbase, to bring the mature miRNA to 21 nt length [101]. This *aMiR* was then cloned into *pK7m24GW,3* downstream of *pSUC2*. The structure of the aMiR was predicted by RNA-fold [102]. Primers used are listed in Table S1. All PCR was performed in a Bio-Rad T100 thermal-cycler using Phusion High-Fidelity DNA Polymerase (Thermo-fisher) or KOD Hot Start DNA Polymerase (Sigma-Aldrich).

### Stable transformation of Arabidopsis

Agrobacterium-mediated Arabidopsis floral dips were used to integrate transgenes as previously described [103], using Agrobacterium strain GV3101. For herbicide selection, plants were grown on ½ MS plates containing 35 μg mL^− 1^ kanamycin or 20 μg mL^− 1^ Basta (Glufosinate-ammonium) and 1% (w/v) sucrose. Alternatively, soil grown plants were sprayed three times with 200 μg mL^− 1^ Basta. Unless otherwise indicated, analysis was conducted on plants homozygous for a given transgene. The *SUC2::FDi* and *FD::FDi* constructs were created in Ler and Col ecotypes, however most analysis was conducted in the Col background to allow comparison to the *fd-5* mutant. To construct the *S- > C* system, *clv3–2* mutants were dipped with the *C* transgene; then, two stable homozygous lines were transformed with *S* (see Fig. S7 for experimental pedigree).

### Flowering time and floral organ phenotypic analyses

For quantitative flowering time analysis, Arabidopsis seeds were stratified (3–4 days at 4 °C) prior to sowing and thinned to uniformity. For each growth experiment, comparisons were made within the same chamber with all groups randomly distributed. Flowering time was determined as the number of days before floral buds were first discerned in the rosette, and rosette leaf number includes all true leaves except those initiated from axillary meristems. To score floral organ number, the number of petals or anthers per flower were counted for nine flowers from plants at the same developmental stage. Individual flower organ counts were averaged to create floral organ counts for individuals. Since floral petal number varied the most between *S- > C 9–3* ‘clv,’ *S- > C 10–5* ‘clv’ and *clv3–2* plants, only petals were counted from different lineages within the *S- > C 9–3* background. For *S- > C* ‘clv’ segregation ratios, seedlings were germinated on ½ MS plates containing 1% (w/v) sucrose before transplantation to soil, to avoid bias for seedling vigor during thinning. Seeds germinated on plates were sterilized through sequential washes of 0.05% Triton-X in 70% ethanol, and two changes of 100% ethanol. Rosette leaf area was calculated via ImageJ. All statistical analysis was conducted using R (V3.4.1).

### DNA extraction and genotyping

To extract genomic DNA, leaf tissue was frozen in liquid nitrogen, ground to a powder and resuspended in buffer consisting of 200 mM Tris-HCL, 250 mm NaCl, 25 mM EDTA and 0.5% (w/v) SDS (pH 7.5). The supernatant was removed, and DNA was subsequently precipitated with isopropanol, pelleted, and washed twice with 70% ethanol, and resuspended in water. Following *SUC2:FDi* crosses, PCR genotyping for the transgene or *fd-5* T-DNA insertion was conducted using the primers and conditions listed in Table S2. Plants homozygous for *hen1–6*, *hst-6*, *hst-15*, or *rdr6–15* were identified via phenotype. PCR was conducted using GoTaq® Green Master Mix (Promega) in a Bio-Rad T100 thermal cycler.

### RNA extraction and qPCR expression analysis

Apical regions, including developing leaves and some petiole, of 15-day old Arabidopsis seedlings were sampled and immediately flash frozen in liquid nitrogen. This apically-enriched tissue was pooled from five plants to produce an independent replicate. RNA was extracted with TRIzol (Invitrogen), per manufacturer instructions. RNA was treated with DNAse I (Thermo-fisher) and subsequently cleaned up via phenol/chloroform extraction and ethanol precipitation. RNA quality was determined via agarose gel electrophoresis and RNA purity and quantity was measured via NanoDrop 2000C (Thermo Scientific). One μg total RNA was used in iScript cDNA Synthesis (Bio-Rad). After confirming primer specificity and efficiency via a standard curve, qPCR measurement of transcript abundance was done using SsoAdvanced Universal SYBR Green Supermix (Bio-Rad) on an Applied Biosystems 7300 real-time PCR system. qPCR primers are listed in Table S3. The *CLV3* qPCR primer facilitated measurement of both transgenic and endogenous *CLV3* transcripts. For all qPCR experiments, 3 technical replicates and 3–6 biological replicates were used. To normalize expression to the amount of meristem derived cDNA in the total cDNA pool, *KNAT1* expression was used as a meristem-specific endogenous control in addition to the β*-tubulin* gene. Importantly, *KNAT1* expression showed no expression trends within the groups sampled, implying its expression was unchanged across the genotypes sampled and serves as an accurate measure of meristem content (Fig. S17). The 2^–ΔΔCt^ method was used to determine expression across groups [104]. Due to the mild difference in *FD* expression, this qPCR experiment was repeated 3 times independently, all of which exhibited the same trend in *FD* expression.

### 5-Azacytidine treatments

Arabidopsis seeds were sterilized (see plant growth conditions) and plated upon ½ MS plates with or without 100 μM 5-Aza, which has been previously demonstrated to reduce genome-wide DNA methylation [105]. Seeds were dark stratified for 3 days at 4 °C before being transferred to light. After 8 days of growth all seedlings were transferred to soil and grown to maturity.

### Sectioning, GUS staining and microscopy

Whole seedlings were cleared in 70% ethanol at room temperature over several days. GUS staining was carried out as previously described [106]. For sectioned tissues, GUS-stained tissues were counter stained with Eosin Y, embedded in paraffin wax, and sectioned (Leica RM2665) into 8 μm ribbons. These ribbons were placed on poly-l-lysine coated slides, re-hydrated, and viewed via light microscopy (Leica DMLS2). GFP fluorescence was viewed via epifluorescence microscopy (Leica MZFLIII). To image Arabidopsis SAMs, the inflorescence tissue was hand dissected before being placed fresh into an environmental scanning electron microscope (Hitachi TM-1000).

## Supplementary Information

**Additional file 1.**

## Data Availability

All data supporting the findings of this study are available within the paper and within its supplementary materials published online. Plant materials used herein will be shared upon reasonable request. All mutants were received from the Arabidopsis Biological Resource Center, which permits their use for academic purposes.

## Abbreviations

5-Aza: 5-Azacytidine
aMiR: Artificial miRNA
AGO: ARGONAUTE
GUS:GFP: β-GLUCURONIDASE:GREEN FLUORESCENT PROTEIN
CLV3: CLAVATA3
Col: Columbia-0
DCL: DICER-LIKE
diRNA: Double-stranded break-induced small RNA
FT: FLOWERING LOCUS T
HST: HASTY
HEN1: HUA ENHANCER 1
Ler: Landsberg *erecta*
LD: Long-day
nt: Nucleotide
PTGS: Post transcriptional gene silencing
qPCR: Quantitative real-time PCR
RDR: RNA-DEPENDENT RNA POLYMERASE
RdDM: RNA directed DNA methylation
SAM: Shoot apical meristem
sRNA: Small RNA
SUC2: SUCROSE-PROTON SYMPORTER2
TGS: Transcriptional gene silencing
Wt: Wildtype
