# Supplementary material for: Plant gene silencing signals move from the phloem to influence gene expression in shoot apical meristems
Source: BMC Plant Biol. 2022 Dec 23;22:606. doi: 10.1186/s12870-022-03998-8 (PMC9783409; doi:10.1186/s12870-022-03998-8)
Supplement: Supplementary file 1 — Additional file 1. [file 12870_2022_3998_MOESM1_ESM.docx]

**Supplemental Material:**

**
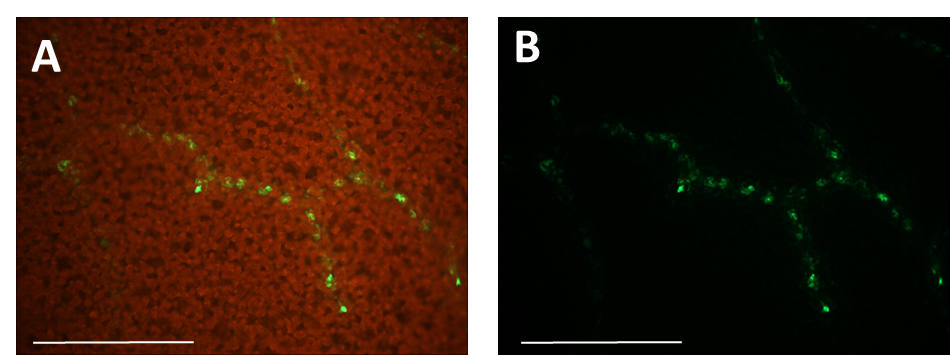
**

**Fig. S1. GFP fluorescence pattern in a mature rosette leaf from a *pSUC2::GUS:GFP* plant.** GFP fluorescence was likewise constricted to the vasculature as with (**A**) or without (**B**) chlorophyll autofluorescence. Scale bar denotes 100 μm.

**
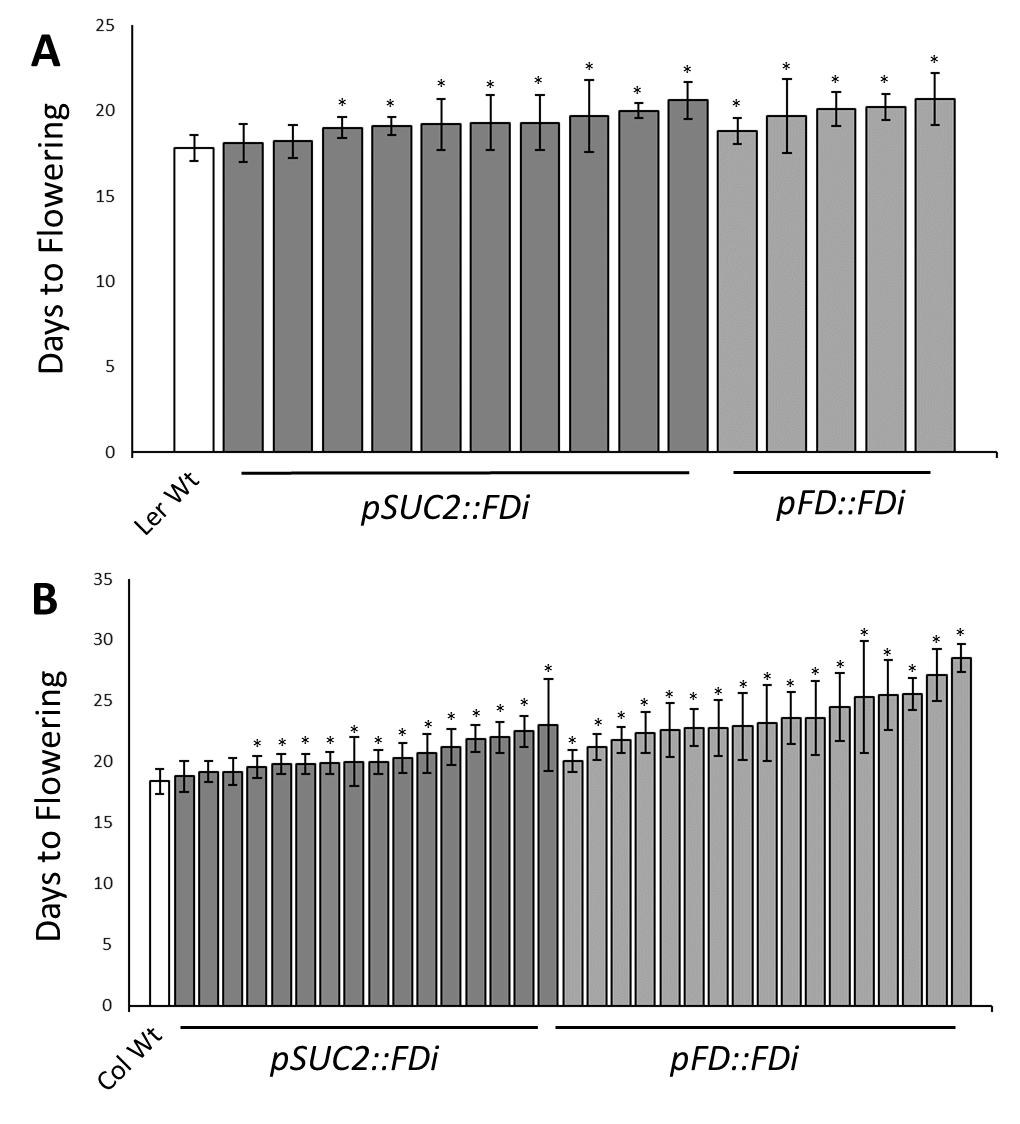
**

**Fig. S2. Under LD photoperiods, T2 *pSUC2::FDi* and *pFD::FDi* lines exhibited delayed flowering in both (A) Ler and (B) Col backgrounds.** Each bar represents the flowering time of an independent insertion event. T2 plants were confirmed to contain the Kanamycin resistance marker. Significant (p>0.05; t-test) differences between transgenic families and their respective wild types are denoted by a *. Error bars represent +/- standard deviation.

**
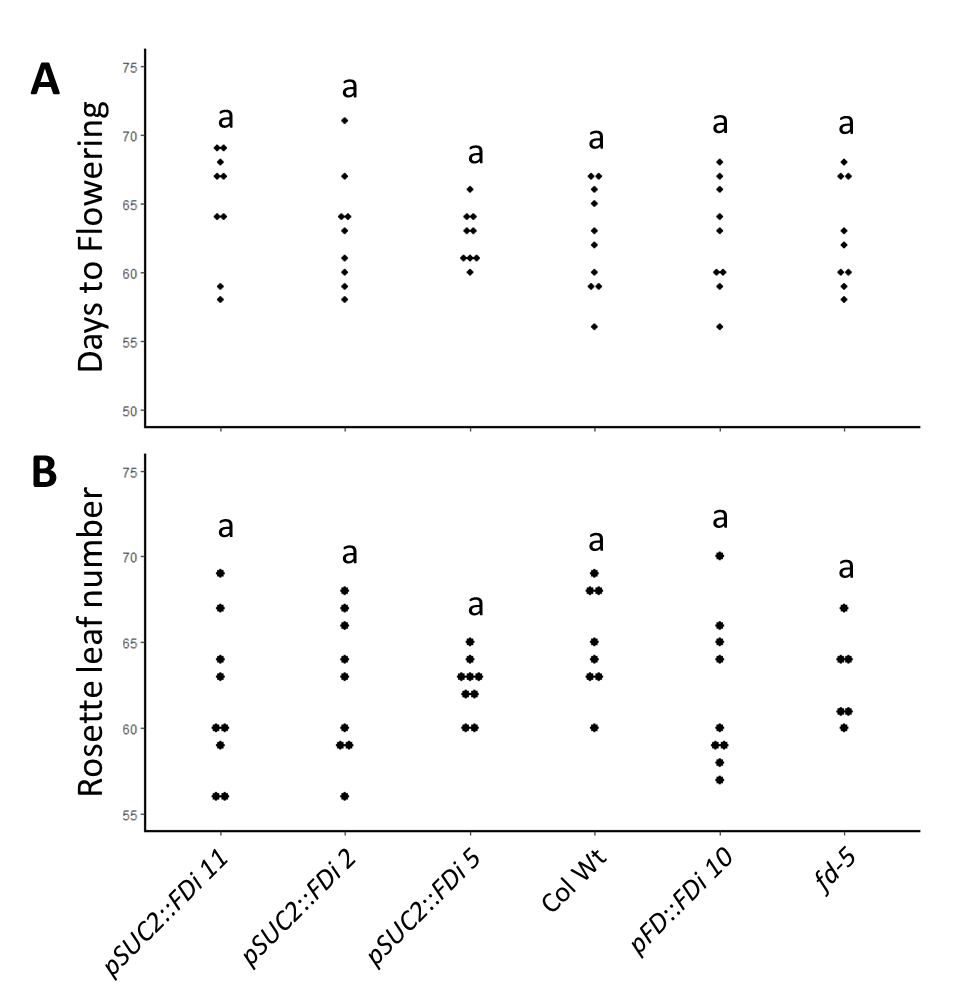
**

**Fig. S3. Under SD photoperiods, *pSUC2::Fdi, pFD::FDi* and *fd-5* lines exhibited flowering time equivalent to Col Wt.** (**A**) All genotypes flowered at a similar day after planting and (**B**) produced an equivalent number of rosette leaves. Each dot represents an individual. Letters denote statistically similar (p>0.05) groups, as determined by ANOVA with post hoc Tukey’s HSD test.


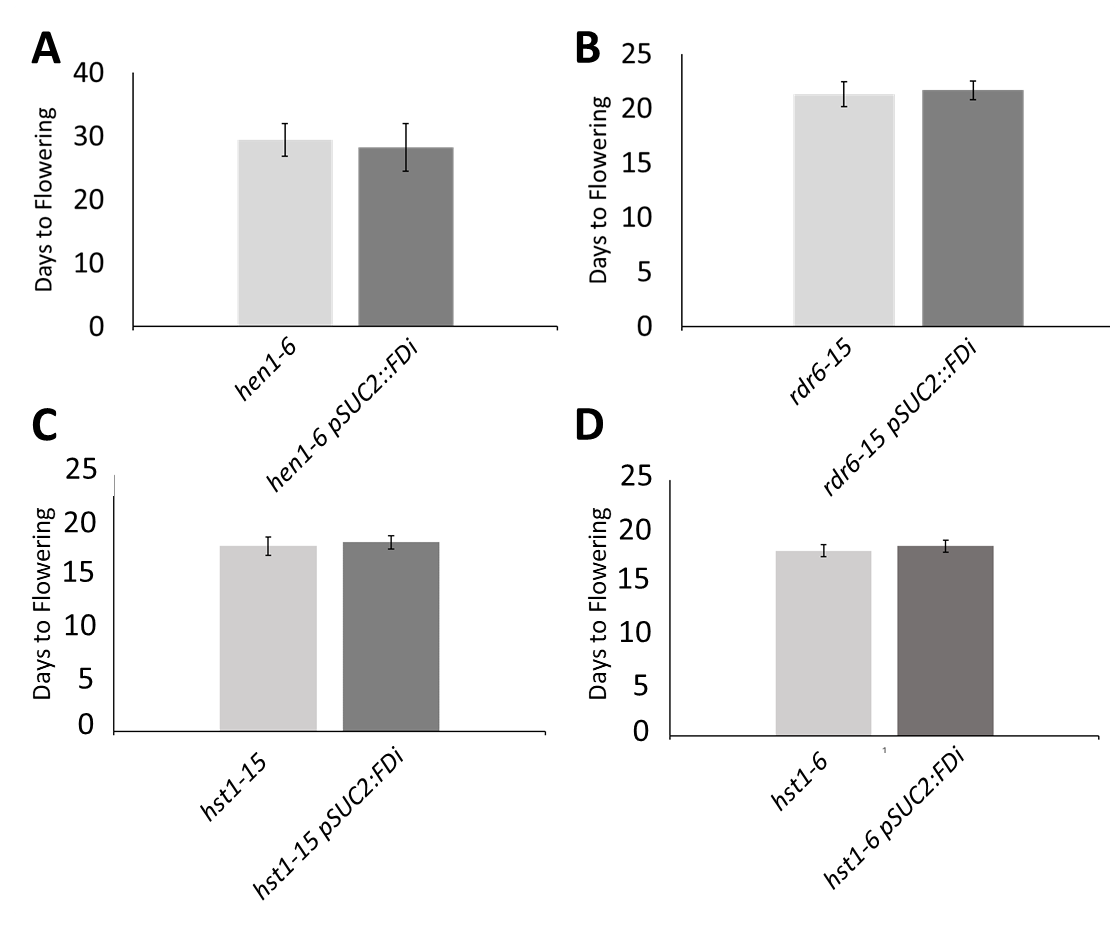


**Fig. S4 The long-day floral delays caused by *pSUC2::FDi* is disrupted when combined with known mutants deficient in sRNA related pathways.** The *pSUC2::FDi* long-day floral delay was abolished when combined with (**A**) *hen1*, (**B**) *rdr6*, or (**C&D**) two *hst* alleles. Pairwise comparisons within all mutant backgrounds illustrated that presence of the *pSUC2::FDi* transgene had no significant (p>0.05;t-test) effect on days to flowering. Error bars denote +/- standard deviation.

**
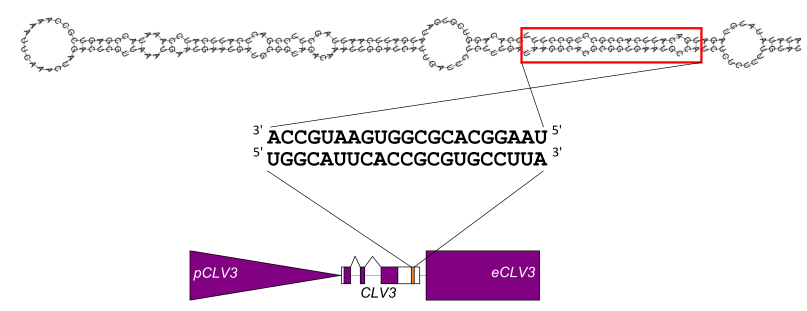
**

**Fig. S5 Depiction of the secondary structure of the *S aMiR* hairpin as well as the base pairing between the mature *aMiR* and the *C* target site.** The RNA-fold predicted structure (top) of the *aMiR* highlights the mature and passenger strand *aMmuMiR124* sequences (outlined in red; *aMmuMiR124* mature sequence is depicted on the bottom strand). The base-pairing between *S* mature *aMiR* and the *C* transgenic target is 100% complementary.


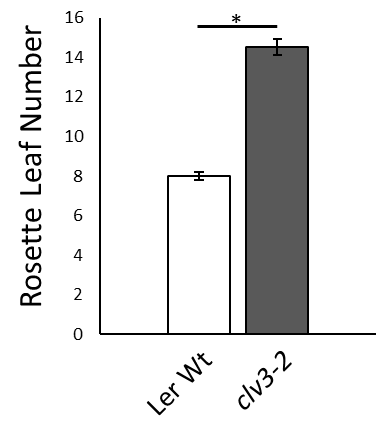


**Fig. S6 Loss of function *clv3-2* mutants produce more rosette leaves before flowering**. An asterisk denotes p values <0.05 from pair-wise t-tests. Error bars denote +/- standard error.


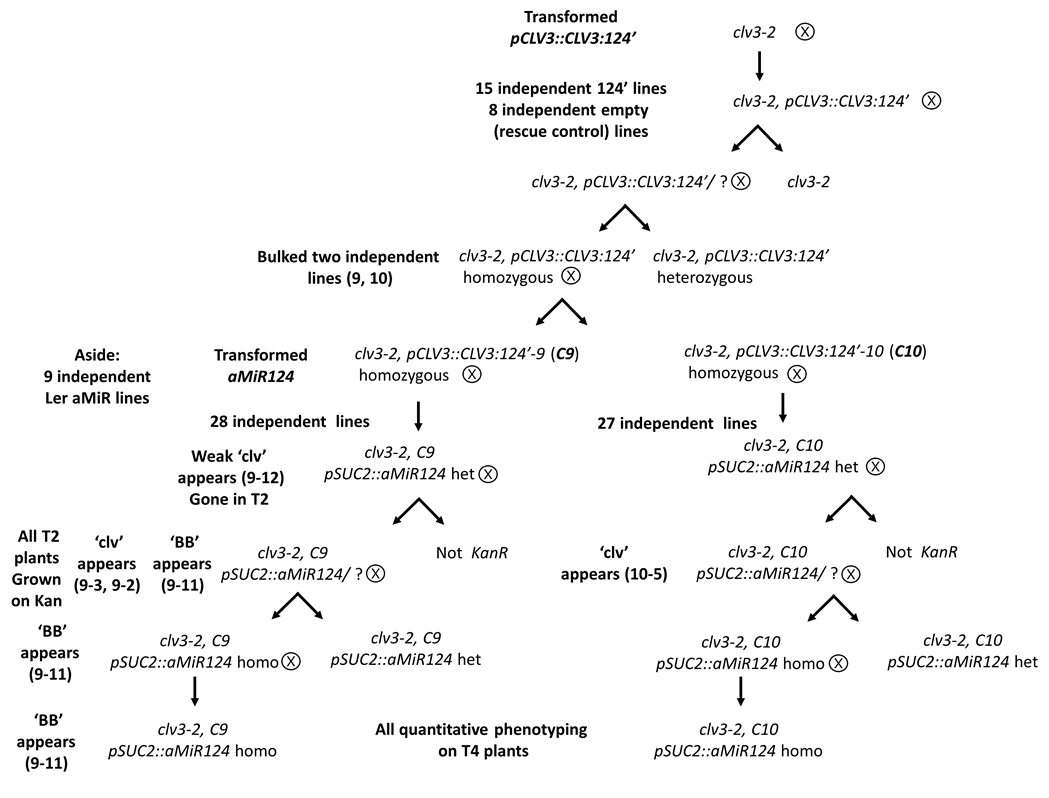


**Fig. S7 *S->C* experiment pedigree.** First, *clv3-2* mutants were transformed with the *pCLV3::CLV3:124*’ (abbreviated to *C*) transgene as well as a rescue control of the *C* cassette lacking the *MmuMiR124’* site. As expected, Eight rescue controls and all 15 *C* lines rescued the *clv3-2* phenotype. From these *C* lines, two independent lines (*C9* & *C10*) were selected on the basis of phenotype stability. *pSUC2::aMiR124 (*abbreviated to S*)* was then transformed into these lines, as well as into the Ler background alone. 28 and 27 independent lines were recovered in the *C9* and *C10* backgrounds respectively. During the generation of homozygous lines, ‘clv’ plants first appeared in the T2 generation. One other T2 line, *S->C 9-11*, produced enlarged flowers with more and larger petals and sepals (big buds, demarked with a ‘BB’ in the figure), but levels of carpels and anthers similar to Ler. This BB line continued to segregate in more advanced generations. Quantitative comparison between lines first took place on T4 homozygous lines.


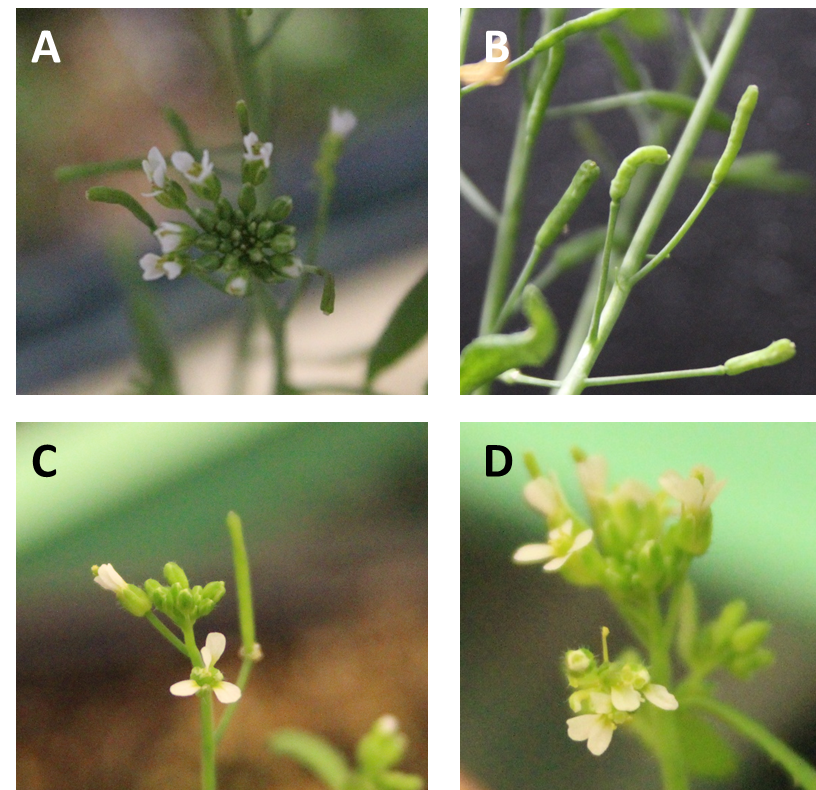


**Fig. S8 Representative images of the minor shoot changes observed in independent T1 *S->C* lines**. *S->C* T1 plants often displayed (**A&B**) mishappen siliques, (**C**) aberrant petal arrangement or (**D**) termination of inflorescences into a determinate floral structure.


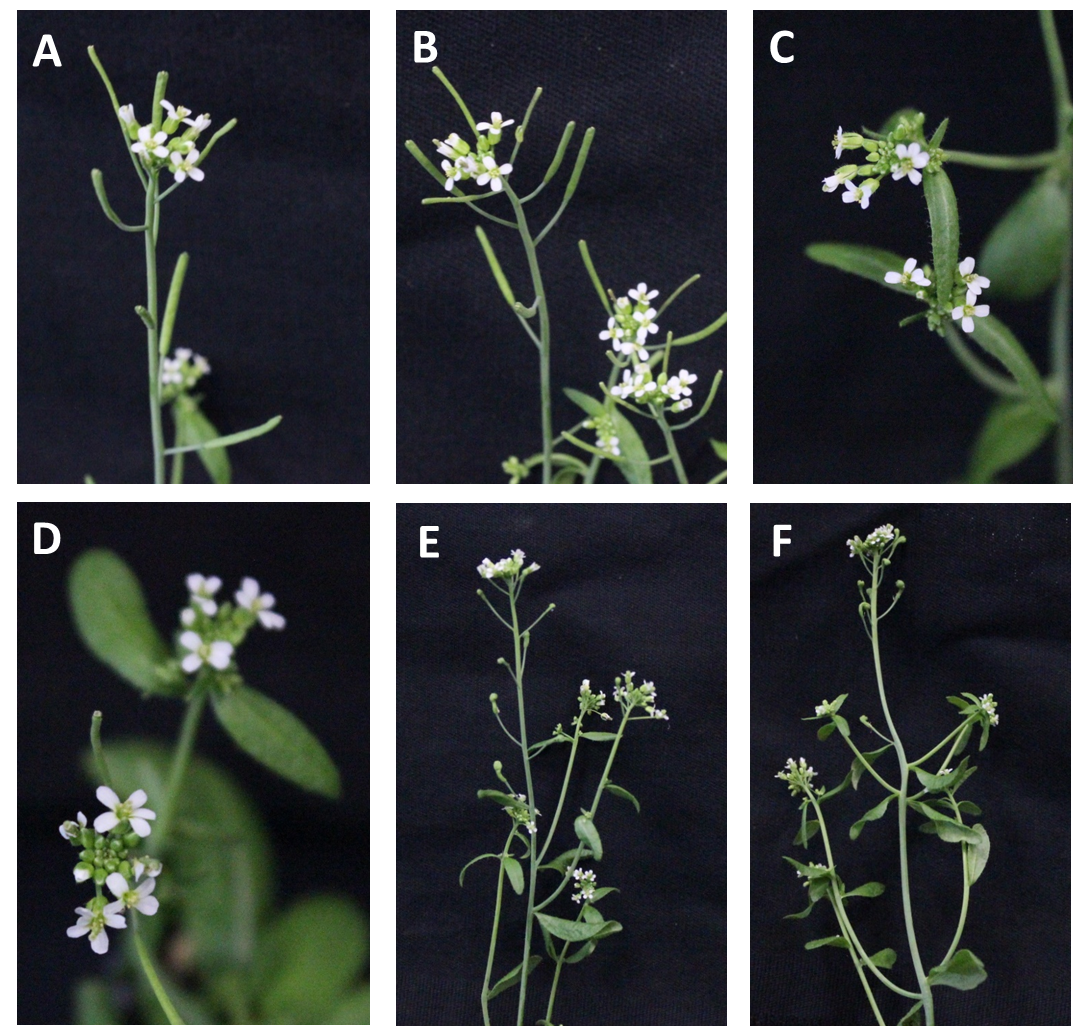


**Fig. S9 Representative images of the shoot changed observed in independent T2 *S->C* lines.** The (**A**) *C10* or (**B**) *C9* rescue lines produced plants that are phenotypically very close the ‘Wt’. (**C&D**) Most *S->C* lines produced a very similar ‘Wt’ phenotype, only occasionally producing flowers with extra petals. However, (**E**) *S->C 9-3* and (**F**) *S->C 10-5* lines produced strong ‘clv’ individuals. One other line, *S->C 9-2*, was later found to likewise produce ‘clv’ individuals (not depicted here). All plants displayed contained the Kanamycin resistance marker lined to *S*.


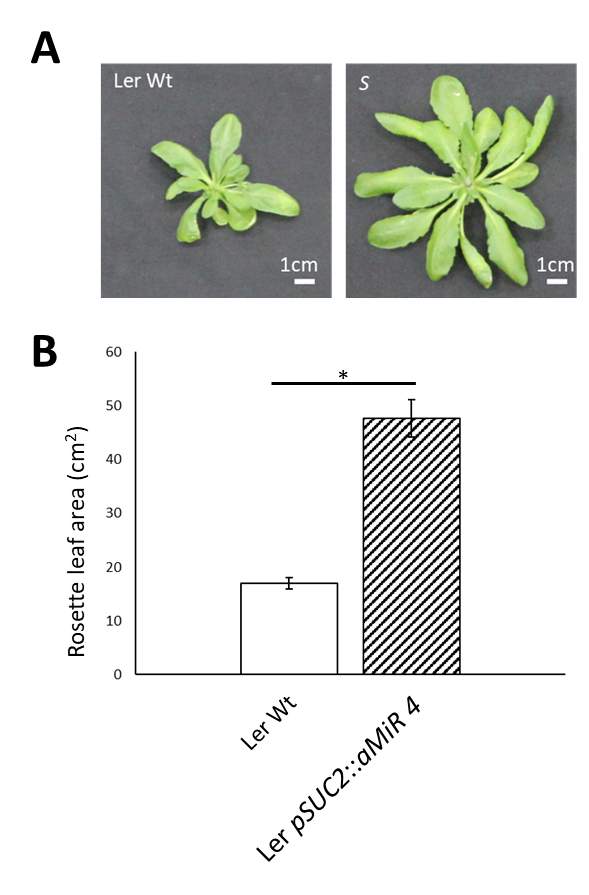


**Fig. S10 Transformation of *S* into Ler Wt produced larger and serrated rosette** **leaves.** (**A**) Representative image of a 35-day old Ler Wt (Left) and an *S* line in the Ler background (right). (**B**) *S* transgenics produced larger rosette areas when compared. A similar change in rosette leaves was seen in *S->C* lines after the transformation of *S*. An asterisk denotes p values <0.05 from pair-wise t-tests. Error bars denote +/- standard error.


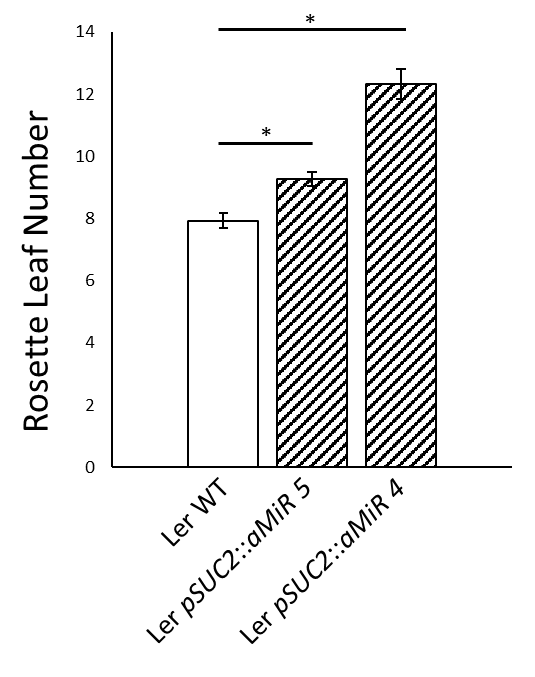


**Fig. S11 *S* plants produced more rosette leaves than Ler Wt.** A similar change in rosette leaf number was seen in *S->C* lines after the transformation of *S*. An asterisk denotes p values <0.05 from pair-wise t-tests. Error bars denote +/- standard error.


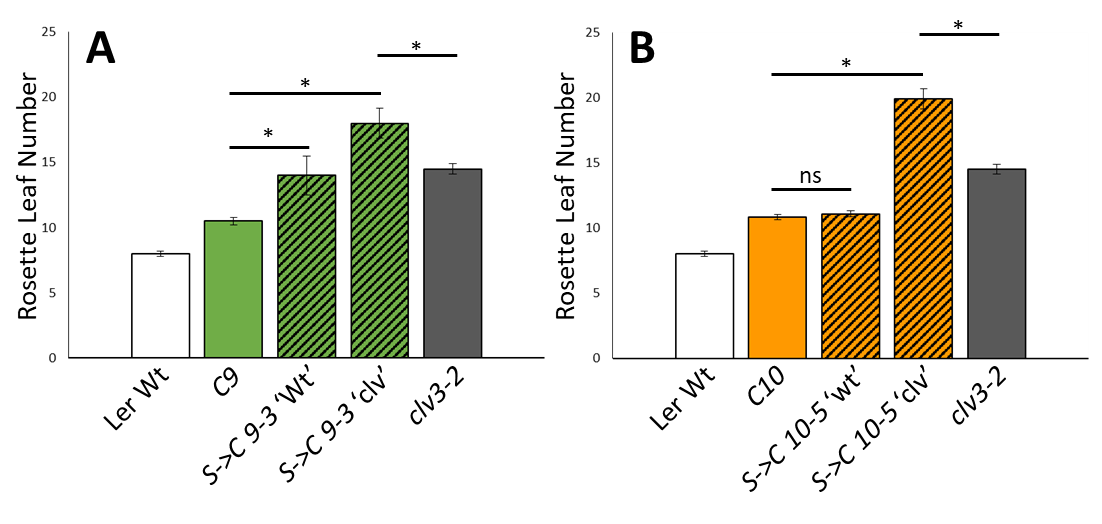


**Fig. S12 The increase in rosette leaf number caused by *S* and ‘clv’ appeared additive in *S->C* plants**. (**A**) *S->C 9-3* ‘Wt’ plants produced more leaves than *C9*, and *S->C 9-3* ‘clv’ plants produced more leaves than *C9* or *clv3-2.* (**B**) *S->C 10-5* ‘clv’ plants produced more leaves than *C10* or *clv3-2.* However, *S->C 10-5* ‘Wt’ plants did not produce more leaves than *C10* in this grow out, consistent with *S* variably impacting rosette leaf number*.* An asterisk denotes p values <0.05 from pair-wise t-tests. Error bars denote +/- standard error.


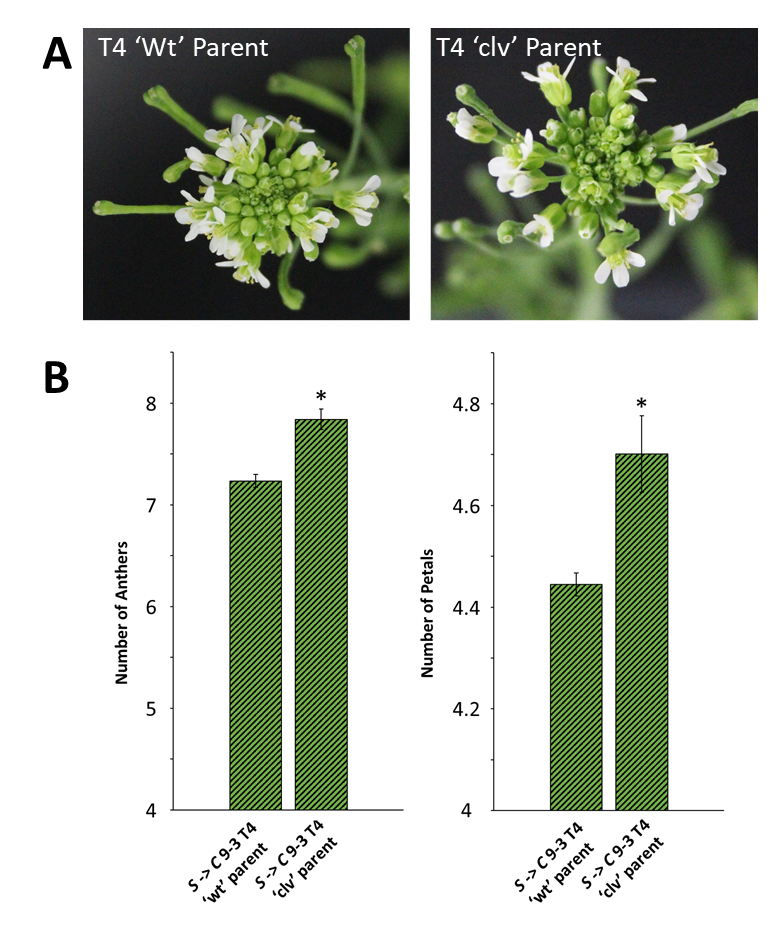


**Fig. S13 *S->C 9-3* transgenics consistently produce intra-family differences ‘clv’ phenotype severity.** (**A**) A second comparison of *S->C 9-3* families confirmed the qualitative differences in silique and apex morphology seen previously. (**B**) Pairwise comparison of the number of petals and anthers produced per flower was consistent with the *S->C 9-3* T4 family from a ‘clv’ parent was more severe than that produced from a separate ‘Wt’ parent. All plants are homozygous for both *S* and *C*. Significant (p>0.05; t-test) differences between families are denoted by a *. Error bars represent +/- standard error.


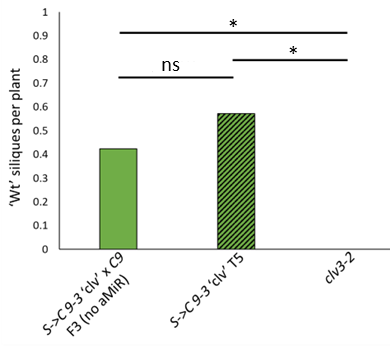


**Fig. S14 No ‘Wt’ siliques were seen on *clv3-2* plants and the number of ‘Wt’ siliques per plant was similar between related plants, regardless of the presence of *S***. An asterisk denotes p values <0.05 from exact Poisson tests (n values are 33, 35 and 33 for *S->C 9-3* ‘clv’ X *C9* F3, *S->C 9-3* ‘clv’ T5, and *clv3-2* respectively). The *S->C 9-3* ‘clv’ x *C9* F3 family measured no longer segregated ‘clv’ plants and retained no copy of *S*.

**
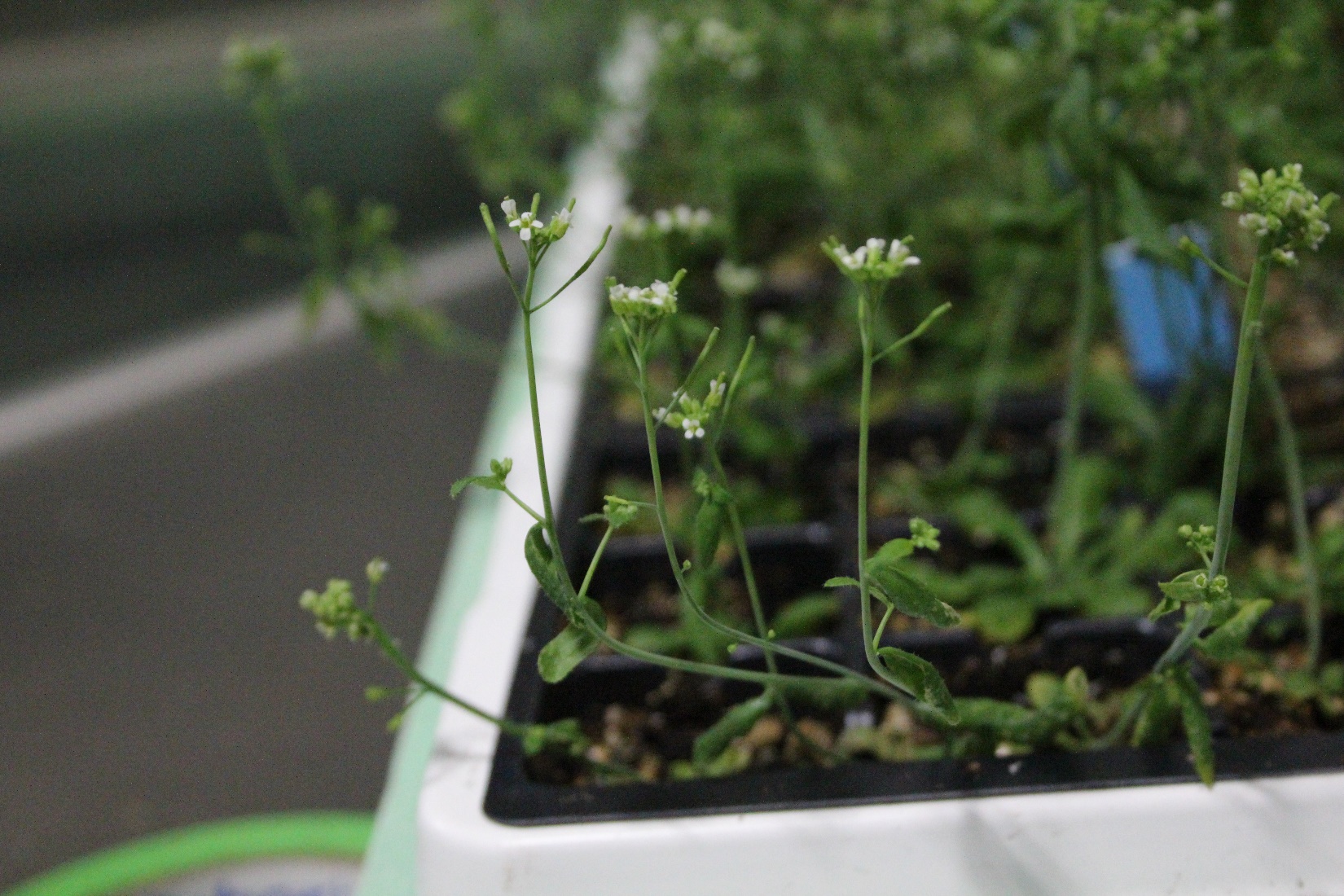
**

**Fig. S15 5-Azacytidine treatment of *9-3 S->C* ‘clv’ plants produced one whole plant reversion.**  This plant produced several sterile flowers before producing straight ‘Wt’ siliques. This event was rare, with only one reversion plant out of 719 5-Azacytidine treated *9-3 S->C* ‘clv’ seedlings.


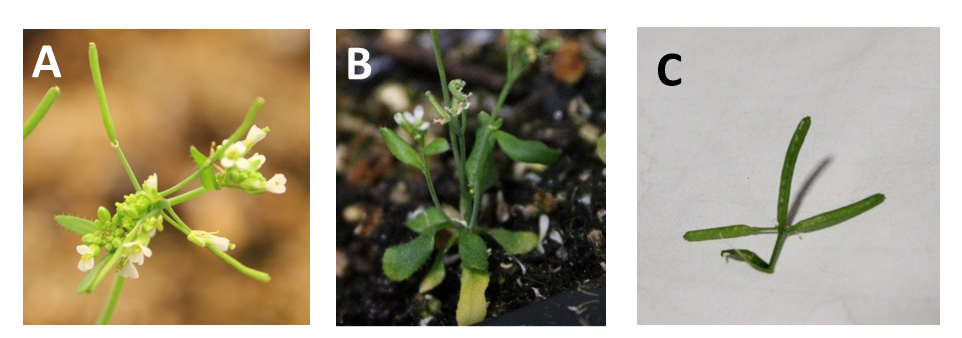


**Fig. S16 *S->C* plants can produce terminal inflorescences.** These inflorescences were observed in various lines including (**A**) *S->C 9* T1 (**B**) *S->C 9-11* ‘Wt’ X *S->C 9-11* ‘BB’ F2 and (**C**) *S->C 9-3* ‘clv’ X *C9* F3 plants.


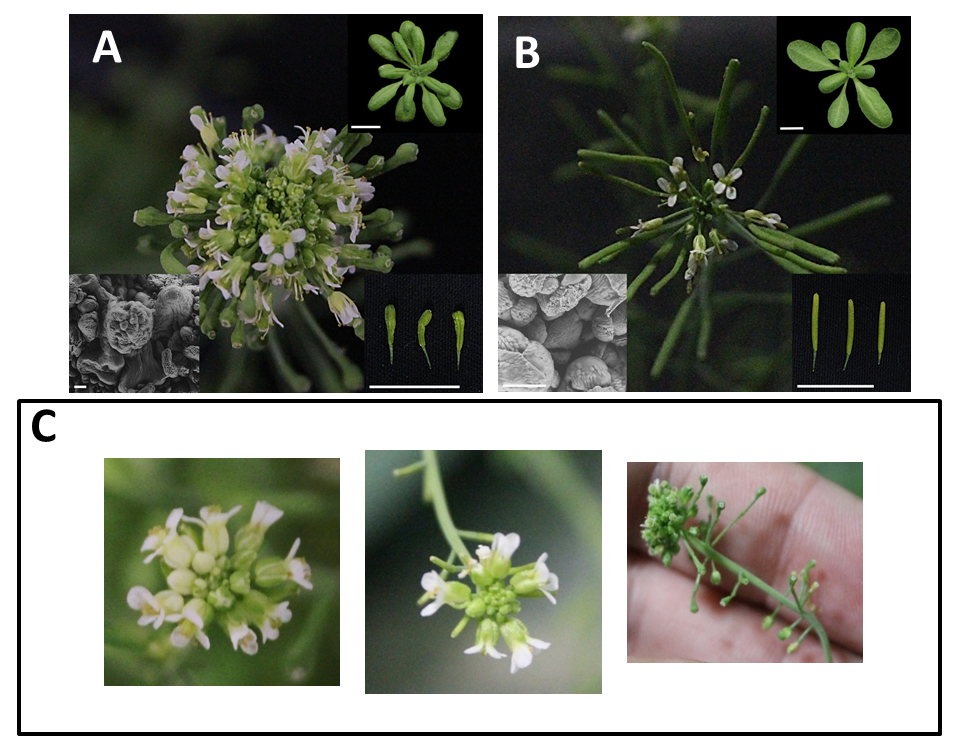


**Fig. S17 *S->C 9-3* ‘clv’ X *C9* F2 plants produced variable shoot phenotypes.** (**A**) *S->C 9-3* ‘clv’ plants were crossed with (**B**) the *C9* progenitor line. The F1 plants appeared phenotypically ‘Wt.’ (**C**) The F2 progeny produced variable shoot morphologies that were not seen in either parent including (left to right) albino sectors, silique sterility and dwarf siliques. Two other crosses likewise produced an F2 population with variable shoot phenotypes. Starting in the top right and rotating clockwise, scale bars represent 1cm, 1cm and100μm.


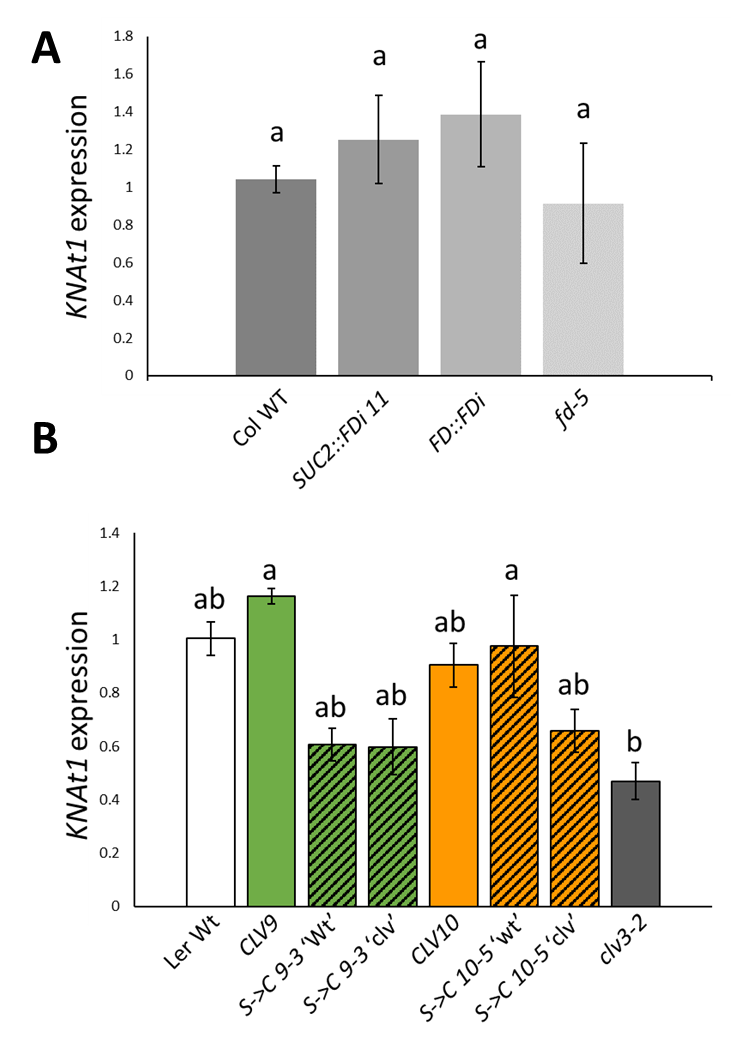


**Fig. S18 qPCR indicated that *KNAT1* did not show any expression trend within any of the transgenic groups**. *KNAT1* expression in (**A**) *pSUC2:FDi* and related lines, or (**B**) *S->C* and related lines. There were *KNAT1* expression differences in B**.** However, no trend was consistently observed for either constructs or plant phenotypes. We suspect instead, these differences correspond to the incorporation of more non-SAM tissue in the sample; that is these expression differences were caused by the meristem making up a varied portion of the sampled apices. Indeed, this was the original purpose of normalizing to a meristem specific marker. Letters denote statistically similar (p>0.05) groups, as determined by ANOVA with post hoc Tukey’s HSD test. Error bars denote standard error. *KNAT1* expression was normalized to *Tubulin.*

**Table S1 Primers used in the construction of long-distance silencing constructs**

| Name | Sequence (5’ to 3’) | Usage |
| --- | --- | --- |
| FD X-F (XhoI-F) | TTCTCGAGGACATCAACCTTGCTTCCATCC | *FDi* fragment cloning into *pHANN* (sense and anti-sense) |
| FD K-R (KpnI-R) | TTGGTACCGGAATTCAAGCTCAGAACAGTTG | *FDi* fragment cloning into *pHANN* (sense and anti-sense) |
| FD B-F (BamHI-F) | TTGGATCCGACATCAACCTTGCTTCCATCC | *FDi* fragment cloning into *pHANN* (sense and anti-sense) |
| FD C-R (ClaI-R) | GCATCGATGGAATTCAAGCTCAGAACAGTTG | *FDi* fragment cloning into *pHANN* (sense and anti-sense) |
| Hann B5F | GGGGACAACTTTGTATACAAAAGTTGGGAAGTTCATTTCATTTGGAGAGG | *FDi* inverted repeat Gateway tagging and cloning into *pDONR221 P5P2* |
| term B2-R | GGGGACCACTTTGTACAAGAAAGCTGGGTAATTTAGGTGACACTATAGAATATG | *FDi* inverted repeat Gateway tagging and cloning into *pDONR221 P5P2* |
| pFD B1-F | GGGGACAAGTTTGTACAAAAAAGCAGGCTAATAGTTATCCAAGGCCCTCTCTACTTG | *FD* promoter Gateway tagging and cloning into *pDONR221 P1P5r* |
| pFD B5r-R | GGGGACAACTTTTGTATACAAAGTTGTTGGAAAAGAGAACAGAAGTGAACCAAC | *FD* promoter Gateway tagging and cloning into *pDONR221 P1P5r* |
| SUC2 B1-F | GGGGACAAGTTTGTACAAAAAAGCAGGCTTGTAAAATCTGGTTTCATATTAATTTC | *SUC2* promoter Gateway tagging and cloning into *pDONR221 P1P5r* |
| SUC2 B5r-R | GGGGACAACTTTTGTATACAAAGTTGTATTTGACAAACCAAGAAAGTAAG | *SUC2* promoter Gateway tagging and cloning into *pDONR221 P1P5r* |
| SacI_pSUC2_Fp | GAAAGAGCTCTAAAATCTGGTTTCATATTAATTTCACACACCAAGTTAC | *SUC2* promoter restriction enzyme cloning into *pK7m24GW,3* |
| NcoI_XhoI_SUC2_Rp | TCAAACCATGGAAGGAACTCGAGATTTGACAAACCAAGAAAGTAAGAAAAAAAAGAAATTTCTTTGAG | *SUC2* promoter restriction enzyme cloning into *pK7m24GW,3* |
| XhoI_mi124_Fp | AGAAACTCGAGCAAAAAAGCAGGCTCAAACACACGCTCG | *aMiR124* restriction enzyme cloning into *pK7m24GW,3* |
| NcoI_mi124_Rp | CAAACCATGGGTACAAGAAAGCTGGGTCATGGCGATGC | *aMiR124* restriction enzyme cloning into *pK7m24GW,3* |
| CLV3aFp | AAAGGATCCGAGCTCTTCGTGGACTTGGAGTTGATGC | *CLV3* genomic fragment 1 amplification and restriction enzyme cloning into *pUC19* |
| CLV3aRp | AACTGCAGACCGGTAAACAGTTGTTGAACTGGACCGG | *CLV3* genomic fragment 1 amplification and restriction enzyme cloning into *pUC19* |
| CLV3bFp | TTTGGATCCACCGGTCTGTTTCATTGCTTTAGTTGTCACG | *CLV3* genomic fragment 2 amplification and restriction enzyme cloning into *pUC19* |
| CLV3bRp | AACTGCAGGATATCCTTTATTGGTTAGTATAGGTGAATGG | *CLV3* genomic fragment 2 amplification and restriction enzyme cloning into *pUC19* |
| CLV124_Duplex | CTAAGGCACGCGGTGAATGCCAGTGCA | Oligonucleotide duplex formation and cloning of the *aMiR124* complementary site into the *CLV3* genomic fragment 2 |
| CLV124_Duplex* | CTGGCATTCACCGCGTGCCTTAGTGCA | Oligonucleotide duplex formation and cloning of the *aMiR124* complementary site into the *CLV3* genomic fragment 2 |

**Table S2 *fd-5* and *pSUC2::FDi* genotyping primers**

| Name | Sequence (5’ to 3’) |
| --- | --- |
| FD-5F | GCTAAGCATCAGAGAAACCATAGACTCTCTGC |
| FD-5R | GGTGGATGGAAGCAAGGTTGATGTCATTCC |
| LBb1.3 | ATTTTGCCGATTTCGGAAC |
| FDiRev | GGTTCCTGGTTCAAAGATCC |
| SUC2F | CCACCACTACAACCACCGC |

**Table S3 qPCR primers used to quantify expression of *FD* and *CLV3***

| Name | Sequence |
| --- | --- |
| qRT FD-F | CTTTTCCACCTCCTGCAACTG |
| qRT FD-R | CATTTTCTGCCTGCAAGTGAG |
| qRT CLV3-F | GTTCAAGGACTTTCCAACCGCAAGATGAT |
| qRT CLV3-R | CCTTCTCTGCTTCTCCATTTGCTCCAACC |
| qRT KNAT1-F | TCCCATTCACATCCTCAACAATC |
| qRT KNAT1-R | CCCCTCCGCTGTTATTCTCT |
| qRT AtBTub-F | CTCAAGAGGTTCTCAGCAGTA |
| qRT AtBTub-R | TCACCTTCTTCATCCGCAGTT |
